# Early‐life foraging: Behavioral responses of newly fledged albatrosses to environmental conditions

**DOI:** 10.1002/ece3.3210

**Published:** 2017-07-26

**Authors:** Sophie de Grissac, Frederic Bartumeus, Sam L. Cox, Henri Weimerskirch

**Affiliations:** ^1^ Centre d'Etudes Biologiques de Chizé CNRS & Université de La Rochelle Villiers en Bois France; ^2^ Centre for Advanced Studies of Blanes (CEAB‐CSIC) Girona Spain; ^3^ CREAF Barcelona Spain; ^4^ Institució Catalana de Recerca i Estudis Avançats (ICREA) Barcelona Spain

**Keywords:** *Diomedea exulans*, ecology, juveniles, learning, seabirds, tracking

## Abstract

In order to survive and later recruit into a population, juvenile animals need to acquire resources through the use of innate and/or learnt behaviors in an environment new to them. For far‐ranging marine species, such as the wandering albatross *Diomedea exulans*, this is particularly challenging as individuals need to be able to rapidly adapt and optimize their movement strategies in response to the highly dynamic and heterogeneous nature of their open‐ocean pelagic habitats. Critical to this is the development and flexibility of dispersal and exploratory behaviors. Here, we examine the movements of eight juvenile wandering albatrosses, tracked using GPS/Argos satellite transmitters for eight months following fledging, and compare these to the trajectories of 17 adults to assess differences and similarities in behavioral strategies through time. Behavioral clustering algorithms (Expectation Maximization binary Clustering) were combined with multinomial regression analyses to investigate changes in behavioral mode probabilities over time, and how these may be influenced by variations in day duration and in biophysical oceanographic conditions. We found that juveniles appeared to quickly acquire the same large‐scale behavioral strategies as those employed by adults, although generally more time was spent resting at night. Moreover, individuals were able to detect and exploit specific oceanographic features in a manner similar to that observed in adults. Together, the results of this study suggest that while shortly after fledging juvenile wandering albatrosses are able to employ similar foraging strategies to those observed in adults, additional skills need to be acquired during the immature period before the efficiency of these behaviors matches that of adults.

## 1 INTRODUCTION

To survive, animals must either be familiar with their surrounding environment or, when facing unfamiliar conditions, able to learn and remember when and where resources can be found, alongside where they can hide from predators. In addition, a good knowledge of their environment may also influence the outcome of competitive interactions (Krebs, 1982; Sandell & Smith, 1991; Stamps, 1987). As such, the efficiency of exploratory behaviors has important consequences for individual survival (Baker, 1993; Verbeek, Drent, & Wiepkema, 1994), and thus individual and population fitness. This is particularly true for immature animals, which are foraging independently for the first time with little to no parental guidance. These individuals typically forage in an unknown environment, and thus rely on an innate ability to find and handle resources (Akesson & Weimerskirch, 2005; Alerstam, Hedenström, & Åkesson, 2003) alongside/or associations with congeners that they can copy (Fagan, Cantrell, Cosner, Mueller, & Noble, 2012; Mueller, O'Hara, Converse, Urbanek, & Fagan, 2013). Moreover, when compared to older individuals, inexperienced juveniles are generally less able to effectively apply crucial skills such as navigation, foraging, and predator avoidance (Marchetti & Price, 1989; Sergio et al., 2014; Thorup, Alerstam, Hake, & Kjellén, 2003). As such, their survival probabilities are usually much lower than those of adults (Clobert, Perrins, McCleery, & Gosler, 1988; Magrath, 1991; Naef‐Daenzer, Widmer, & Nuber, 2001; Perrin, 1979). In addition, they may be more vulnerable to sudden changes in habitat availability (e.g., anthropogenic disturbance and prey depletion/redistribution) alongside extreme climatic events (Nevoux, Weimerskirch, & Barbraud, 2007). As such, innate abilities for orientation and foraging cannot be the only mechanisms juveniles rely on to survive, and a certain amount of learning and adjustment is probably necessary in order to endure the critical period of early life and later recruit into a population.

In many seabird species, chicks are left alone at the nest by their parents before fledging. As such, they have to leave the colony and forage at sea independently without the opportunity to learn from their parents. Vital foraging skills are therefore likely learned quickly (within the first few months at sea). A large capacity for behavioral adaptation to environmental variability may also aid survival. Although immaturity can last several years (e.g., up to ten years for albatrosses), these first months at sea appear particularly critical to the survival of juvenile seabirds (Daunt, Afanasyev, Adam, Croxall, & Wanless, 2007; Horswill et al., 2014; Riotte‐Lambert & Weimerskirch, 2013). However, despite this, little is currently known about the detailed foraging tactics of animals during this time, alongside how individuals respond to environmental cues that aid in the acquisition of resources (Hazen et al., 2012; Lewison et al., 2012; Scales et al., 2014). In particular, there is a lack of quantitative analyses examining how juveniles survive, which is likely because observing young seabirds at sea is challenging.

Long‐ranging pelagic seabirds, such as albatrosses and petrels, forage on heterogeneously distributed prey in an environment with little physical constraints and a paucity of landmarks (Weimerskirch, 2007; Weimerskirch, Gault, & Cherel, 2005). Moreover, they may rely on particular environmental conditions to be efficient. For example, albatross and petrel flight is strongly influenced by wind, which is used to minimize corresponding energetic costs (Felicísimo, Muñoz, & González‐Solis, 2008; Pennycuick, 1982; Weimerskirch, Guionnet, Martin, Shaffer, & Costa, 2000). In addition, individuals from these species may forage at specific habitats, such as regions of elevated productivity, shelf slopes, ocean fronts, or oceanic waters with species‐specific temperature preferences where prey availability is enhanced (Hunt, 1991; Kappes, 2009; Louzao et al., 2011; Pinaud & Weimerskirch, 2007; Scales et al., 2016). However, the use of specific wind conditions and oceanographic habitat features by inexperienced juvenile albatrosses and petrels to optimize energy acquisition is unclear. Specifically, it is unknown whether individuals forage using strategies similar to those employed by older, more experienced adults as soon as they fledge, or whether they progressively acquire these with time.

The aim of this study was to examine the early foraging behaviors of juvenile wandering albatrosses *Diomedea exulans* during their first months at sea following fledging. We use a dataset of eight juveniles, tracked using GPS/Argos transmitters during their first eight months after leaving their natal island in the southern Indian Ocean, and compare this to the tracks of 17 adults from the same colony. Through the use of a behavioral clustering algorithm, we identify the main behavioral modes adopted by these animals when at sea and examine (1) whether the at‐sea behaviors of juveniles change through time after fledging, (2) to what extent behavioral mode is influenced by external conditions (e.g., light, oceanographic features, and climatic factors), and (3) whether juvenile behavioral modes differ from those of adults.

## 2 METHODS

### 2.1 Fieldwork and telemetry

Fieldwork was conducted on the Crozet Islands, Southern Indian Ocean (46.2°S, 52.4°E), during the breeding seasons of 2013 (juveniles and adults), and 2014 and 2015 (for adults only). In 2013, 10 wandering albatross chicks (six males and four females), that were about to fledge, were fitted with GPS/Argos satellite transmitters (Platform Terminal Transmitter, PTT 100, Microwave Telemetry, Columbia, USA). These were attached to the central back feathers using adhesive tape (TESA^®^) and glue (Loctite^®^). GPS tags were programmed to record one location every two hours (accuracy ~20–75 m). These locations were then transmitted every three days by the Argos transmitters (which were powered by solar battery). The device weighed 65 g which is <1% of the average mass of the juvenile birds (10.3 ± 3.0 kg) and well below the limit recommended for flying birds (Phillips, Xavier, Croxall, & Burger, 2003). Individuals were sexed using a molecular sexing method (Weimerskirch, Lallemand, & Martin, 2005).

To compare juvenile behavior with that of adults, 17 breeding adults from the same colony were tracked with GPS transmitters during incubation from January to March (2013–2015 inclusive). This time period corresponds to the first three months of juvenile independence following fledging in late December. Adults were equipped with GPS tags using the methods described above. GPS tags recorded one location every 15 min. To be comparable to the data retrieved from juveniles, locations were later resampled at 2 hourly intervals.

### 2.2 Clustering of foraging behaviors

Expectation Maximization binary Clustering (EMbC; Garriga, Palmer, Oltra, & Bartumeus, 2016) was used to classify the behavioral modes adopted by an individual while at sea. The EMbC algorithm is a variant of Gaussian Mixture Model maximum‐likelihood estimation (or Expectation Maximum Clustering). It is a robust, nonsupervised multivariate clustering algorithm that considers the correlation and uncertainty of variables to give a meaningful local label that can be easily biologically interpreted. This includes a percentage uncertainty for each classification. Behaviors were categorized using two input variables: the velocity and turning angle of a bird between each location. Both parameters were calculated with loxodromic distances and bearings using the “geosphere” R package (Hijmans, 2015). Tracks were clustered into four behavioral categories: high velocity/low turning angle (HL), high velocity/high turning angle (HH), low velocity/low turning angle (LL) and low velocity/high turning angle (LH; Table 1 and detailed statistics in Table S1). HL and HH behaviors correspond to rapid movements which we have, respectively, termed “ballistic” (commuting phases, i.e., rapid speed and high directionality) and “diffusive” (sinuous exploratory phases at large scale using looping movements). LL and LH behaviors correspond to slow movements when the bird is mainly sitting on the water, and are referred to, respectively, as “resting” (bird drifting passively) and “active sitting.” The later of these, “active sitting,” reflects a mixture of different behavioral types, such as (1) the use of a sit‐and‐wait foraging strategy (Weimerskirch, Cherel, Cuenot‐Chaillet, & Ridoux, 1997), (2) intensive foraging on a prey patch using short flights interspersed with sitting bouts, and (3) the transition between the “resting” mode and one of the two rapid modes (“ballistic” and “diffusive”; i.e., when a bird takes off with a change of direction at the end of the two hours segment). These interpretations have been validated in earlier papers through the use of visual observations, activity logger analyses, and energetic budget analyses (Louzao, Weigand, Bartumeus, & Weimerskirch, 2014; Weimerskirch, Delord, Guitteaud, Phillips, & Pinet, 2015; Weimerskirch, Pinaud, Pawlowski, & Bost, 2007; Weimerskirch et al., 1997, 2002). In the analyses performed here, we used only track portions labeled with 100% certainty by the algorithm (i.e., when the time interval used to compute velocity and turns was not superior to two hr).

**Table 1:**
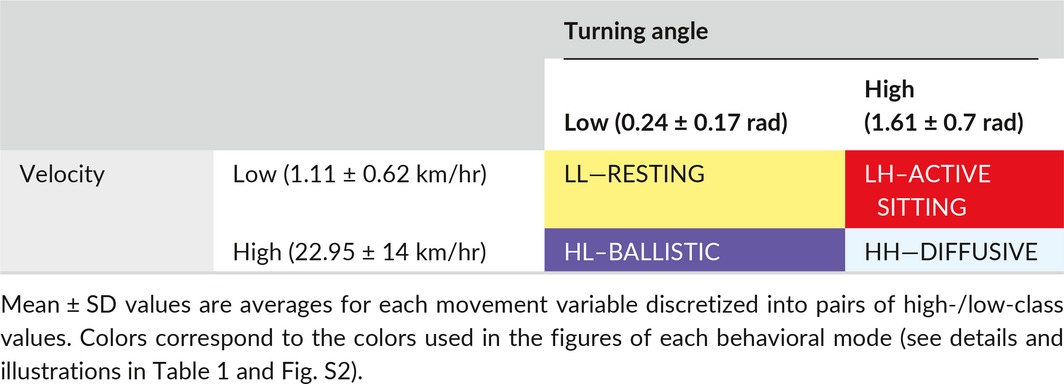
Binary clusters as determined by the EMbC algorithm (Garriga et al., 2016) and their corresponding behavioral mode. Adapted from Louzao et al. (2014)

### 2.3 Environmental variables

To study the relationship between behavioral mode and biophysical environmental conditions, we considered several environmental variables that are known to influence the foraging behaviors of marine predators elsewhere (Friedland et al., 2012; Hunt, 1991; Hunt et al., 1999; Hyrenbach, Veit, Weimerskirch, & Hunt, 2006; Louzao et al., 2011). These were as follows: six hourly wind velocity and direction (0.25° resolution), bathymetry (0.016° resolution), monthly chlorophyll‐α concentration (CHLa, 0.04° resolution), daily sea‐level height anomaly (SLHA, 0.25° resolution), and moon brightness. Bathymetry, wind, and CHLa data were downloaded from the NOAA coast watch website (http://coastwatch.pfeg.noaa.gov). The SLHA data were taken from the Aviso data portal and were produced by Ssalto/Duacs with support from CNES (http://www.aviso.altimetry.fr/duacs/). Moon data were obtained via the “lunar” R package (Lazaridis, 2014) and used to compute a continuous moon brightness index by associating the height of the moon above the horizon with lunar phase. To aid interpretation, this index was also split into three categories corresponding to (1) “dark nights” when the moon was under the horizon and/or in its first quarter, (2) “half‐moon nights” when the moon was above the horizon and between its first and third quarter, and (3) “full‐moon nights” when the moon was above the horizon and in its third quarter.

### 2.4 Statistical analyses

All juvenile analyses excluded the first 15 days of a track following departure from the colony; as during this period, individuals are known to drift on the water while they wait for favorable wind conditions, upon which adopt a specific directional flight in order to rapidly move away from their natal ground and reach lower latitudes (De Grissac, Börger, Guitteaud, & Weimerskirch, 2016; Weimerskirch, Akesson, & Pinaud, 2006). Moreover, only the first eight months of data received from an individual was used, even if transmission were received beyond this. First, we used linear mixed models, Cochran–Mantel–Haenszel tests, and chi‐squared tests to ascertain how behavioral mode proportions of adults and juveniles varied with sex, through time and in comparison with each other. Then, to investigate how environmental conditions influence the probability for juveniles and adults to use the different behavioral modes, we performed a set of multinomial logistic regressions using the R package “mlogit” (Croissant, 2013). These models predict the probability that an individual engages in each of the four behaviors (see descriptions above) according to the corresponding environmental conditions of each location (Awkerman, Fukuda, Higuchi, & Anderson, 2005; Freeman et al., 2013). We also included day/night duration in our analyses, which was calculated using the “geosphere” R package (Hijmans, 2015). However, it is noted that this parameter is correlated with time, as birds were tracked from summer (December/January) to winter (July). Predictions are made in turn for each environmental variable at each time sample, while all other variables are considered fixed at average values. The model then tests how these predicted probabilities vary with respect to each other when environmental variables change (e.g., how the probability of one behavior changes compared to the probability of another, according to the value of environmental variable). Four models were fitted using four subsets of data: (1) diurnal locations of juveniles, (2) diurnal locations of adults, (3) nocturnal locations of juveniles, and (4) nocturnal locations of adults. This was because the behaviors of individuals differed substantially between the day and night (see Results section). Model selection was performed using Akaike's Information Criterion (AIC), by giving preference to the model exhibiting the lowest AIC. To avoid over‐parametrization, when the change in AIC was <2, we examined the number of parameters comprising a model and favored models with a smaller number of variables. Environmental variables were interpreted as having a significant influence on behavioral mode probabilities at *p* < .05. Autocorrelation in model residuals was tested for and found minimal at lag of 1–3 (see Fig. S2). To our knowledge, there is currently no way to account for autocorrelation in multinomial regression. For juvenile models, we got rid of most of the autocorrelation (at least to a lag 1) by resampling the input data to remove one of two consecutive locations. We did not resample at a courser resolution as this would have resulted in sample sizes too small for robust statistics, which is also the reason adult data were not resampled. Nevertheless, selected juvenile models with and without this correction produced very similar results. This suggests that while autocorrelation may be a source of some bias in our analyses, models are likely robust enough to give reliable results.

All values are given as average ±1 *SD*, unless stated. All analyses were computed using the R Software Environment (R Core Team 2015).

## 3 RESULTS

From the juvenile dataset, one track was discarded because just after leaving the colony the individual landed on the sea and drifted slowly for one month before the device stopped transmitting. This suggests the bird died before starting any foraging movements. A second track was discarded because device malfunction resulted in multiple oversized gaps between locations (up to three weeks in length). As such, the following results correspond to analyses conducted on the eight remaining tracks (five males and three females). Transmissions were received for between 112 and 372 days (mean = 246.5 ± 88.6 days), yielding an average of 10.8 ± 0.8 GPS locations per day and a dataset comprising 18,457 locations in total. Two individuals were tracked for <4 month (one male and one female) while the other six birds were tracked for a total of eight months or more (up until late December 2014). Across the three years (2013 through to 2015), adults were tracked between January and March for time periods lasting 8–25 days, resulting in a dataset comprising 4,385 locations in total.

### 3.1 Overall movements

**Figure 1:**
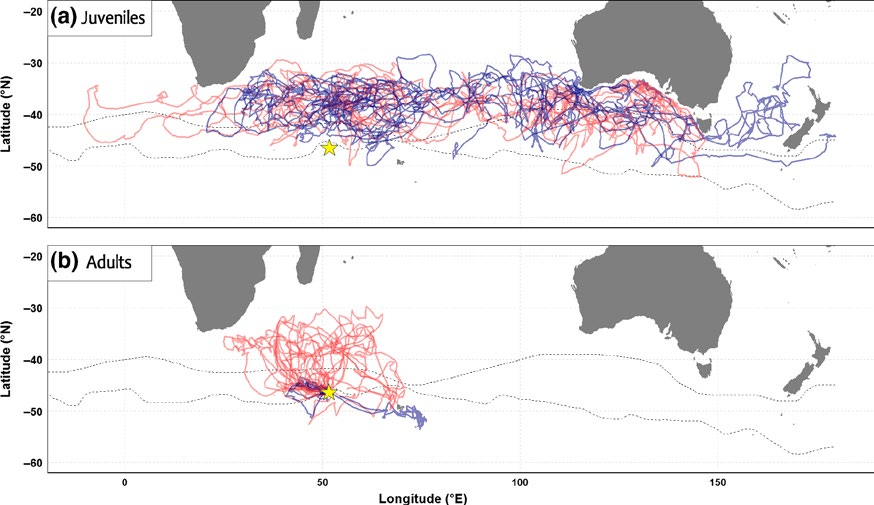
Trajectories of the eight juvenile (a) and 17 breeding adult (b) wandering albatrosses. Males are marked in blue and females in red. Crozet Island is symbolized by the yellow star. Dashed lines are, from South to North, the sub‐Antarctic front and the southern subtropical front

**Figure 2:**
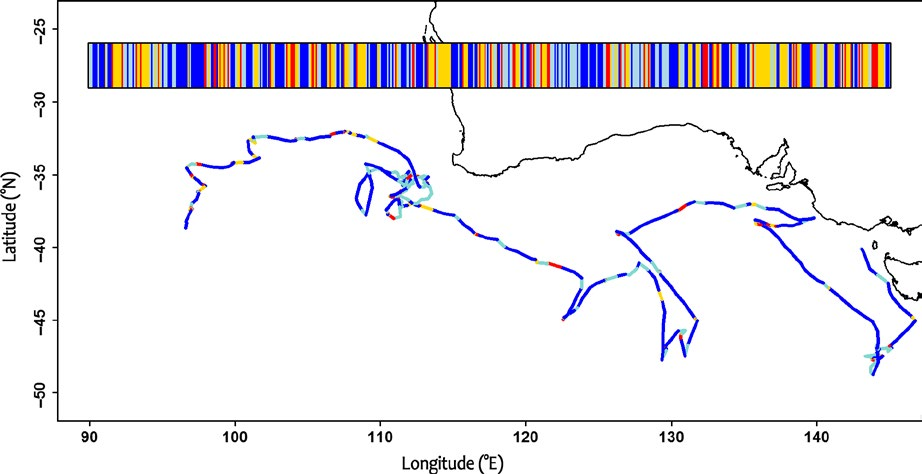
Fifty‐day portions of a juvenile's trajectory with behavioral modes as segmented by EMbC. The bar at the top of the figure shows an ethogram of behavioral segmentations (yellow: resting; red: active sitting; blue: ballistic movement; light blue: diffusive movement), while the central map shows an example juvenile track colored (as described above) by behavioral mode)

During their first year at sea, juveniles dispersed widely across the subtropical Indian Ocean (40–25°S), ranging from the South African to Australian coasts and the Tasman Sea. Two individuals entered the Pacific Ocean and one the Atlantic Ocean (Figure 1). Until up to their eighth month at sea, all juveniles remained in relatively warm waters north of the sub‐Antarctic convergence (Figure 1). When in the western part of the Indian Ocean, they remained north of the subtropical front. Individuals flew mainly over oceanic waters but also visited shelf slopes without ever crossing the continental limit (200 m deep; see Fig. S2). There was individual variability in terms of the types of habitats visited, the times at which they were exploited and generalized movement patterns. While some individuals moved from oceanic waters to concentrate around shelf slopes, others remained continuously in oceanic habitats, and tended to use oceanographic features such as ridges and sea mounts and make larger scale movements (see Fig. S3). At a maximum, juveniles reached areas as far as 3,560 ± 1,277 km from the colony during their first month at sea, and after eight months, their maximum range averaged 7,674 ± 2,860 km (max 11,000 km). The six individuals that were tracked over eight months each covered an average total distance of 83,449 ± 7,837 km since their departure (min 74,328 km to max 96,120 km). The daily distances covered by all individuals increased significantly (Kruskal–Wallis: $\chi_{7}^{2}$ = 23.414, *p* = .0014, Tukey post hoc: *p* = .011) between the first and second months from 237 ± 66 to 353 ± 41 km/day, but showed no significant trend afterward (Kruskal–Wallis: $\chi_{6}^{2}$ = 9.794, *p* = .13). This compares to an adult average daily travel distance of 503 ± 134 km. As illustrated in Figure 2, across all tracks, movements were characterized by continuous changes in the four behavioral modes determined by EMbC.

### 3.2 Sex differences

Juvenile diurnal activity was influenced by sex during the first 3 month at sea following fledging (see Fig. S4), with males spending more time resting than females (linear mixed model: *t*_6_ = 2.87, estimate = 9.61 ± 3.34, *p* = .028). However, after 3 months, these differences were no longer significant (*t*_6_ = −1.14, estimate = −4.31 ± 3.76, *p* = .29). Due to the small sample size, the effect of sex was not evaluated for behavioral and environmental analyses on both juvenile and adult datasets.

### 3.3 Influence of diurnal cycle on behavior

The proportion of time spent in each of the four behavioral modes differed between day and night for both juveniles (Cochran–Mantel–Haenszel test controlling for individual: $M_{3}^{2}$ = 2,758.9, *p* < .001, Fig. S1) and adults (Cochran–Mantel–Haenszel test controlling for individual: $M_{3}^{2}$ = 190.9, *p* < .001). During the day, juveniles and adults predominantly performed “rapid” flying behaviors (ballistic: 42.1 ± 5.0% (juveniles), 43.2 ± 11.8% (adults), and diffusive: 33.2% ± 3.7 (juveniles), 31.4 ± 8.3% (adults); see the top of Fig. S1). In contrast, during the night juveniles mainly rested (43.7 ± 7.5%), while adults performed both ballistic (35.1 ± 10.1%) and resting (28.2 ± 7.6%) behaviors (see the bottom of Fig. S1).

### 3.4 Changes with time

**Figure 3:**
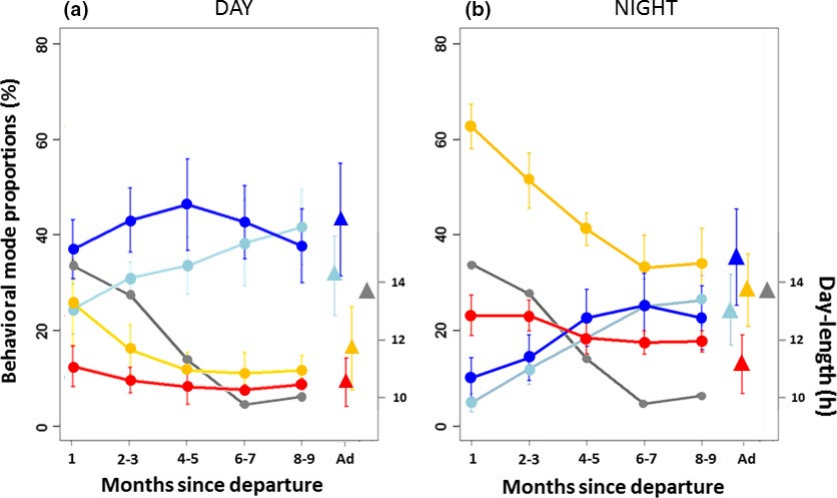
Change over time of the proportion of behavioral mode use by juveniles during day (a) and night (b) alongside comparison with those of adults. Proportions of behavioral mode use (yellow: resting; red: active sitting; blue: ballistic movement; light blue: diffusive movement) are averaged by than across individuals and over periods of 1–2 months for juveniles (dots), and over the whole trip for adults (triangles). Bars show standard deviations. The gray line is the mean day duration in hours along juvenile tracks (from January to August), and the gray triangle is the mean duration of the day during the period across which adult tracking took place (summer)

Juvenile behavioral mode proportions varied with time since departure from the colony, following the same trend for both day and night analyses (Figure 3). Resting proportions decreased, while the use of diffusive movements steadily increased and ballistic behavioral proportions increased and then stabilized. The proportional use of active‐sitting behaviors remained relatively stable throughout the tracking period and was generally lower than that of the other behavioral modes. During the first month, compared to adults, juveniles spent more time resting and less time in flight (chi‐squared test: $\chi_{3}^{2}$ = 84.352, *p* < .001). After this first month, during daylight, juvenile behavioral proportions fell within the range of those observed for adults (chi‐squared test: $\chi_{3}^{2}$ = 1.3607, *p* = .714, Figure 3a). For nighttime behaviors, differences between adults and juveniles were generally larger than those observed during the day, and were maintained up until the eighth month (chi‐squared test over the whole period: $\chi_{3}^{2}$ = 196.12, *p* < .001), although a decrease through time was noted. Overall, across an entire trip (inclusive of both day and night movements), juveniles used resting and active‐sitting modes more often than adults, and ballistic behaviors less often (Figure 3b).

### 3.5 Multinomial logistic regression

All variables retained following model selection had a significant influence on variation in the use probability of at least one behavior with respect to another. These are listed in Table 2, alongside an estimation of their overall contribution (absolute value of the *t*‐statistic). Full model outputs with parameter estimates and significance are displayed in Table 2 of Fig. S4 shows variation in behavioral use probabilities according to each individual variable tested, while keeping all other variables fixed at the mean value observed across the dataset.

**Table 2:** Variables retained after model selection for the four multinomial logistic regression models alongside their overall contribution (absolute value of the *t*‐statistic)

	Day/Night length	Wind speed	Bathymetry	log(CHLa)	SLHA	Moonlight
Day						
Juveniles		**0.84**	0.35	0.83		
Adults		**0.88**	0.55		0.65	
Night						
Juveniles	0.81	0.69	0.41			**1.04**
Adults		**1.09**	0.58	0.56	0.67	0.71

The most important variables are highlighted in bold.

#### 3.5.1 Correlations between behavioral modes and night durations/time

**Figure 4:**
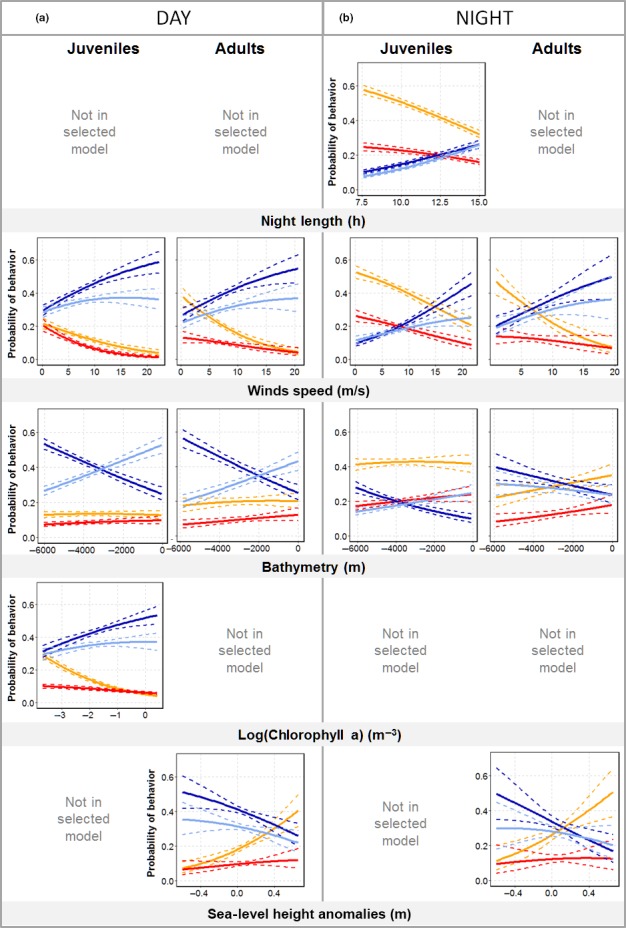
Modeled relationship between environmental variables and the probability of each behavioral mode use from multinomial logistic regression. Columns show day (a) and night (b) for juveniles and adults. Plain lines show predicted probabilities for juveniles and adults to perform each of the four behaviors (yellow: resting; red: active sitting; blue: ballistic movement; light blue: diffusive movement) in response to changes in night length, wind speed, bathymetry, chlorophyll‐a, and sea‐level height anomalies. Dashed lines represent 95% CIs

Night duration significantly influenced the behavioral mode probabilities of juveniles during the night (Figure 4b, top row). With increasing night length, the probabilities of resting and active‐sitting behaviors decreased while there was an increase in the probabilities of using ballistic (coefficient = .2, *p* < .001) and diffusive (coefficient = .27, *p* < .001; Figure 4b, top row) behaviors. However, because night duration and time are highly correlated, it was not possible to determine whether these observations were mainly due to a seasonal decrease in night duration or also because juveniles gained experience through time. Night length varied little during the tracking period of adults. It had no effect on adult behavioral mode probabilities and was not retained following model selection procedures.

#### 3.5.2 Environmental correlates of diurnal behavior

The influences of key environmental variables on the behavioral mode probabilities of juveniles and adults during the day are shown in Figure 4 (see detailed model outputs in Table S2). Wind speed was the most influential of these for adults and juveniles (Table 2). For both groups, with increasing wind speed, individuals were significantly more likely to perform ballistic and diffusive movements in place of active‐sitting or resting behaviors (all coefficients > .25, *p* < .05 for adults and juveniles, see Tables S2 and S3). Individual responses to changes in bathymetry were also similar across both the juvenile and adult groups. Here, with decreasing depth, the probability of performing ballistic movements progressively decreased in favor of more diffusive movements (coefficient = −.42, *p* < .05 [adults]; coefficient = −.29, *p* < .05 [juveniles], see Tables S2 and S3), which became more likely at depths shallower than 3,000 m (juveniles) and 2,000 m (adults). For juveniles, in locations with higher CHLa concentrations, flying (i.e., ballistic and diffusive) behavioral mode probabilities increased compared to resting modes (coefficient = .34 [ballistic] and 0.3 [diffusive], both *p* < .05, see (Tables S2 and S3). However, changes in CHLa concentrations did not influence adult behavioral mode probabilities. SLHAs only influenced adult behavioral mode probabilities. Here, individuals were more likely to engage in flying behaviors compared to resting behaviors when SLHAs were negative (coefficient = .24 [ballistic] and .23 [diffusive], both *p* < .05, see Tables S2 and S3).

#### 3.5.3 Environmental correlates of nocturnal behavior

At night, juveniles performed mainly resting or active‐sitting behaviors. However, they became more likely to fly (i.e., through the performance of ballistic or diffusive movements) as wind speed increased (all *p* < .05, see Tables S2 and S3). Specifically, the probability of flying became higher than that of sitting at the surface when wind speeds exceeded 13 m/s (Figure 4). The same pattern was observed for adults, but with a much lower wind speed threshold of 5 m/s. Bathymetry had a significant effect on juveniles, driving an increase in the probability of using active‐sitting or diffusive behavior instead of ballistic movements at shallower depths (coefficient = .27 [active sitting] and .35 [diffusive], both *p* < .05, Tables S2 and S3). CHLa concentration and SLHA did not influence the nocturnal behaviors of juveniles (Figure 4, Table 2), while SLHA did influence the behaviors of adults during the night. Here, although individuals were more likely to perform ballistic and diffusive movements when over negative SLHA, individual variability was high (Figure 4), and this was only significant when comparing ballistic movement probabilities to resting probabilities (coefficient = −.34, *p* = .05).

**Figure 5:**
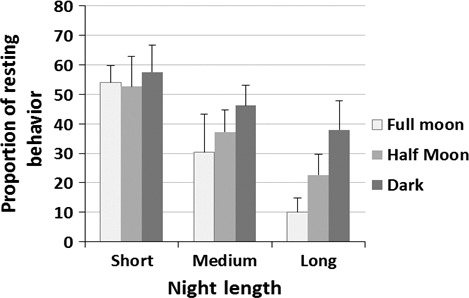
Proportion of resting behavior used by juveniles during full moon, half‐moon and dark nights according to night length. Long nights correspond to durations >13.5 hr, medium nights to durations of between 10.5 and 13.5 hr and short nights to durations <10.5 hr (classes obtained from observations of night length distribution)

Moon brightness strongly influenced the nocturnal behavioral mode probabilities of both juveniles and adults (Table 2). When moonlight intensity increased, individuals were more likely to perform both ballistic and diffusive movements over resting (Table S2 and Fig. S5). We also observed a slight decrease in the probability of active‐sitting behaviors when moonlight was very bright (i.e., during full‐moon nights). For the juveniles tracked across both summer and winter, the effect of moonlight on behavioral mode probabilities is combined with the effect of night duration. During winter, when the nights are the longest (>13.5 hr), the proportion of time spent resting at night during a full moon was similar to that during the day (Figure 5). Conversely, when nights were short (<10.5 hr), juveniles spent most of the night resting regardless of moon brightness.

## 4 DISCUSSION

Following fledging, juvenile wandering albatrosses spend 3–4 years at sea before returning to their natal colony. In this study, we show that within the first year, individuals develop and employ movement strategies similar to those observed in adults. At 2 months, juveniles were already found throughout the subtropical Indian Ocean and had daily travel distances exceeding 300 km, which is close to the average traveling rate of adults (Riotte‐Lambert & Weimerskirch, 2013; Salamolard & Weimerskirch, 1993). At the beginning of their at sea period, these performances may be due to innate capacities that would help respond to changes in self‐internal state and programmed necessities (Akesson & Weimerskirch, 2005; Alerstam et al., 2003). However, the marine environment in which these individuals forage is highly dynamic, and changes in biophysical oceanographic conditions drive the patchy distribution of prey alongside heterogeneity in movement costs for predators (Constable, Nicol, & Strutton, 2003; Hunt et al., 1999; Pennycuick, 1982; Weimerskirch, Guionnet, et al., 2000). As such, to optimize energy acquisition, juveniles need to be able to detect and interpret such structuring, and adapt their search strategies accordingly (Bartumeus & Catalan, 2009; Charnov, 1976). Such abilities are generally thought to be acquired and/or improved upon through time with experience (Newton, 2008). We show that, during the first months of their life at sea, inexperienced juveniles are able to respond to changes in their biophysical environment as adults do.

### 4.1 Individual variability

Juvenile wandering albatrosses displayed high individual variability in habitat use and large‐scale movement patterns. This is similar to that observed in migratory adults, which has been linked to age, colony, and sex (Weimerskirch et al., 2015). Partially inherited factors, such as personality (Patrick & Weimerskirch, 2015; Verbeek et al., 1994) and/or individual quality (i.e., consistent between‐individual differences related to phenotypic characteristics (Wilson & Nussey, 2010), may also influence individual variability in juvenile exploratory movements (Dingemanse, Both, Drent, van Oers, & van Noordwijk, 2002; Dingemanse, Both, Noordwijk, Rutten, & Drent, 2003). Indeed, individuals may differ in how they collect information about their surroundings and react to new environments (Verbeek et al., 1994). This may also be contingent upon early‐life conditions which can further influence individual quality and behavioral choice (Fay, Barbraud, Delord, & Weimerskirch, 2016; Fay, Weimerskirch, Delord, & Barbraud, 2015; Lindström, 1999; Stamps, 1987). However, while individual variability is common across a number of albatross species (Louzao et al., 2014; Shaffer, Costa, & Weimerskirch, 2001), observed levels in this study are likely emphasized due to the relatively small sample size (eight individuals), and so our results should be treated with some degree of caution.

### 4.2 Behavioral changes over time

Despite high individual variability, several behavioral patterns were identified that were consistent across individuals. During their first month at sea, juveniles flew < adults, spending more time on the surface, particularly at night. However, by the second month, daytime juvenile behavioral mode proportions were similar to those of adults. This may be because the flight capacities of juveniles, as indicated by daily travel distances here alongside patterns in speed and wind use elsewhere (Riotte‐Lambert & Weimerskirch, 2013; Weimerskirch et al., 2006), are already almost equal to those of adults. As such, the first month appears to be a period of rapid change for young birds when basic but vital capacities are likely developed through muscle reinforcement and optimal flight practice. This would enable individuals to quickly learn how to effectively use winds to reduce energetic movement costs. Indeed, rapid improvements in flying performance have already been observed for other young seabirds (e.g., brown booby *sula leucogaster* (Yoda, Kohno, & Naito, 2004). As such, long periods spent resting on the water during the first month of the at sea period may reflect especially high levels of flight energy expenditure in birds which at this time are untrained in flying skills. Subsequent decreases in the total time spent foraging alongside the probable use of suboptimal search strategies (Daunt et al., 2007; Wunderle, 1991; Wunderle & Martinez, 1987), mean that during this first month juveniles may rely on energetic reserves accumulated on the nest (Weimerskirch, Barbraud, & Lys, 2000). After the possible depletion of these reserves within the first few weeks at sea, finding food may become a priority (Soutullo, Urios, Ferrer, & Peñarrubia, 2006) which may explain why this adjustment period is so short, and basic foraging skills are rapidly learnt.

With time, juveniles tended to behave more and more similarly to breeding adults, yet after six months at sea still spent comparatively more time resting at night. We suggest this reflects the comparatively higher energetic demands of breeding adults alongside central place constraints, which force consistently higher levels of foraging effort regardless of underlying environmental conditions (Mackley, Phillips, Silk, Wakefield, Afanasyev, Fox, et al., 2010; Mackley, Phillips, Silk, Wakefield, Afanasyev, & Furness 2010; Pinet, Jaeger, Cordier, Potin, & Le Corre, 2011; Salamolard & Weimerskirch, 1993). Indeed, nonbreeding adult wandering albatrosses spend up to 50% more time on the water at night during winter, than the breeding adults of this study do in summer (>60% vs 30%, respectively; Weimerskirch et al. (2015)). Progressive changes in juvenile behavior from the first month until at least the sixth may not solely be a consequence of ontogenetic modification, and could also relate to external constraints such as increases in night duration during winter which may induce observed decreases in the proportion of time spent resting.

Adaptation to light availability by diurnal animals is well known across a range of taxa (including seabirds) which use vision to navigate and locate prey (Clarke, Chopko, & Mackessy, 1996; Dias, Granadeiro, & Catry, 2012; Fernandez‐Duque, 2003; Mackley, Phillips, Silk, Wakefield, Afanasyev, & Furness 2010; Regular, Hedd, & Montevecchi, 2011; Weimerskirch, Gault, et al., 2005). As such, even if many of the prey of albatrosses (e.g., fish and squid) approach the surface at night, the ability to see and capture them from the air in the dark is reduced (Weimerskirch, Gault, et al., 2005). Indeed, direct measures of prey capture events have shown that wandering albatrosses obtain most of their prey during the day. However, they can also feed successfully at night (Weimerskirch, Gault, et al., 2005; Weimerskirch et al., 2007), through the use of a “sit‐and‐wait” foraging strategy (which is likely included in our active‐sitting behavior), has been suggested as an efficient alternative to the forage‐in‐flight strategy, particularly when individuals are highly time constrained (e.g., breeding adults; Louzao et al. (2014)). During such periods, it may be difficult for birds to satisfy energetic demands during daylight hours, and so compensatory night foraging may be required. Such behavior may also be required during winter, when day lengths are reduced. For juveniles, this effect may be exacerbated by suboptimal foraging and/or competitive skills, which can prevent individuals from finding enough food during daytime and so encourage additional night foraging. Indeed, immature (>1 year old) albatrosses have been shown to forage more than adults during the night (Weimerskirch, Gault, et al., 2005), which would also explain why juveniles in our study spent less time resting at night than wintering adults (40% vs the 60% reported by Weimerskirch et al. (2015)). Increases in foraging time to compensate suboptimal foraging performance have been suggested for other juvenile seabirds such as shags *phalacrocorax aristotelis* (Daunt et al., 2007). However unlike albatrosses, shags are unable to forage at night and so juveniles experience lower survival rates in winter when day duration is decreased. Notably, juvenile albatrosses in our study appear to take advantage of periods of increased moon light to forage more at night and compensate for a reduction in day length, thus particularly during winter. Lunar phase is known to influence the behavior of many birds, including pelagic seabirds such as petrels and shearwaters (Pinet et al., 2011; Yamamoto et al., 2008). A slight decrease in active‐sitting behavior probabilities for nights with a full moon may reflect behavioral changes, possibly because diurnally vertically migrating prey may position themselves at greater depths when moon light is increased rendering this foraging strategy less effective (Conners, Hazen, Costa, & Shaffer, 2015; Weimerskirch et al., 1997).

### 4.3 Adjustment to oceanographic conditions

The impact of wind on the flight costs and velocities of adult wandering albatrosses is well documented (Pennycuick, 1982; Weimerskirch, Guionnet, et al., 2000). In this study, juveniles appear to also react strongly to wind conditions, adjusting their behaviors similarly to adults. This suggests juveniles possess innate abilities to exploit wind, although further experience may be required to increase efficiency. For example, Riotte‐Lambert and Weimerskirch (2013) have shown individuals progressively fly mostly with tail and side‐to‐tail winds during the first months. The correct use of wind conditions is key to the efficient management of energetic budgets.

Besides wind and ambient light, young albatrosses also responded to changes in oceanic environmental conditions. When over waters deeper than 3,000 m (i.e., the majority of south western Indian Ocean), individuals were more likely to perform larger scale movements by performing ballistic movements. In contrast, diffusive movements were favored when flying over waters shallower than 3,000 m. Such sinuous movements are typically related to area restricted search behaviors (Kareiva & Odell, 1987; Louzao et al., 2014), which generally occur in areas of higher and more predictable prey density (Weimerskirch et al., 2007). The increased use of these types of strategies by juveniles in shallower waters may reflect the increased presence of favorable foraging habitats such as shelf slopes, seamounts, and ridges, which can aggregate prey (Fauchald & Tveraa, 2003; Louzao et al., 2011; Paiva, Geraldes, Ramírez, Garthe, & Ramos, 2010), which would trigger more intense exploration and/or use of prey search behaviors. In addition, juveniles increased search activity in areas with higher CHLa, which directly links to primary productivity and possibly reflects the idea that this parameter can be used as a proxy of higher‐trophic level prey availability (Hyrenbach et al., 2006). Coupled with detailed observations of bird trajectories, these results reiterate the importance of oceanographic features such as ridges, seamounts, and shelf slopes, alongside cyclonic oceanic eddies (which are known to be associated with increases in primary productivity) to foraging seabirds (Hunt et al., 1999 p. 199; McGillicuddy et al., 2007; Kai et al., 2009; Tosh et al., 2015). Although we did not detect links between adult behavioral patterns and areas of higher CHLa concentration, they have been previously shown to forage extensively over productive shelf slopes and oceanic plateaus (Waugh & Weimerskirch, 2003; Weimerskirch, 2007). Moreover, individuals were more likely to perform flying behaviors where sea‐level anomalies are negative, conditions that are typical at the centre of cyclonic eddies. Overall, juveniles, during their first months at sea, seemed to respond in a similar way to adults to the environment they encountered. In addition, these observed behavioral modifications suggest that juveniles may be able to respond, innately or after a short period of learning, to environmental proximal signals of prey availability such as water color and dimethyl‐sulfide odor alongside the presence of bird aggregations as it has been shown for adults (Fauchald, 2009; Nevitt, 2000). Finally, it is worth noting that juveniles and breeding adults face different constraints (notably, central place constraint for breeding birds), and a lack of nonbreeding adult data limits the interpretations made here.

Although recently fledged juveniles are able to detect and use potentially favorable habitats, they concentrate mainly in oceanic waters, where resources are known to be less abundant (Hunt et al., 1999). It is only after around six to seven months that some individuals start to concentrate around well‐known productive areas such as the shelf‐edges off the southwestern coast of Australia (Great Australian Bight). This may be partially explained by intraspecific and/or interspecific competitive pressures, as juveniles, with lower foraging performances, are thought to be less competitive than more experienced and so may be excluded from the most favorable areas birds (van den Hout et al., 2014; Sol, Santos, Garcia, & Cuadrado, 1998; Wheelwright & Templeton, 2003). However, while juveniles were able to accurately follow shelf‐slope contours (occurring at depths between 2,000 and 200 m), they seem to strictly avoid flying over the actual continental shelf (above 200 m deep) just like adults (Nicholls et al., 2002; Weimerskirch, 2007 and see Fig. S2).

## 5 CONCLUSION

In conclusion, juvenile albatrosses were able to respond to the environmental conditions they encountered during their dispersive movements in a manner similar to that observed in adults. However, it appears they only attain the body conditions and foraging skills of adults after several years of immaturity (Weimerskirch, 1992). Subsequently, the extensive duration of the immaturity period cannot be explained by movement performances and behavioral decisions alone, and may be additionally related to the accumulation of foraging skills, such as optimal prey choice and capture, alongside competitive ability. Such a long time period may also be necessary to acquire the experience needed to be able to forage from a central place (such as the breeding colony) and memorize information about the environmental conditions surrounding breeding grounds (when they begin visiting colonies from around four to five years of age). Such knowledge would enable immatures to bear both the costs of reproduction and maintenance. While current data do not yet enable investigations toward all these aspects of ontogeny of foraging behavior, new developments in tracking technologies will allow future studies to address these unknowns.

## CONFLICT OF INTEREST

None declared.

## DATA ACCESSIBILITY

All tracking data are available in the Dryad public archive at http://datadryad.org/.

## References

[ece33210-bib-0001] Akesson, S. , & Weimerskirch, H. (2005). Albatross long‐distance navigation: Comparing adults and juveniles. Journal of Navigation, 58, 365–373.

[ece33210-bib-0002] Alerstam, T. , Hedenström, A. , & Åkesson, S. (2003). Long‐distance migration: Evolution and determinants. Oikos, 103, 247–260.

[ece33210-bib-0003] Awkerman, J. , Fukuda, A. , Higuchi, H. , & Anderson, D. (2005). Foraging activity and submesoscale habitat use of waved albatrosses *Phoebastria irrorata* during chick‐brooding period. Marine Ecology Progress Series, 291, 289–300.

[ece33210-bib-0004] Baker, R. R. (1993). The function of post‐fledging exploration: A pilot study of three species of passerines ringed in Britain. Ornis Scandinavica, 24, 71–79.

[ece33210-bib-0005] Bartumeus, F. , & Catalan, J. (2009). Optimal search behavior and classic foraging theory. Journal of Physics A: Mathematical and Theoretical, 42, 434002.

[ece33210-bib-0006] Charnov, E. L. (1976). Optimal foraging, the marginal value theorem. Theoretical Population Biology, 9, 129–136. 1273796 10.1016/0040-5809(76)90040-x

[ece33210-bib-0007] Clarke, J. A. , Chopko, J. T. , & Mackessy, S. P. (1996). The effect of moonlight on activity patterns of adult and juvenile prairie rattlesnakes (*Crotalus viridis viridis*). Journal of Herpetology, 30, 192–197.

[ece33210-bib-0008] Clobert, J. , Perrins, C. M. , McCleery, R. H. , & Gosler, A. G. (1988). Survival rate in the great tit *Parus major* in relation to sex, age, and immigration status. The Journal of Animal Ecology, 57, 287–306.

[ece33210-bib-0009] Conners, M. G. , Hazen, E. L. , Costa, D. P. , & Shaffer, S. A. (2015). Shadowed by scale: Subtle behavioral niche partitioning in two sympatric, tropical breeding albatross species. Movement Ecology, 3, 28. 26392862 10.1186/s40462-015-0060-7 PMC4576409

[ece33210-bib-0010] Constable, A. J. , Nicol, S. , & Strutton, P. G. (2003). Southern ocean productivity in relation to spatial and temporal variation in the physical environment. Journal of Geophysical Research: Oceans, 108, 8079.

[ece33210-bib-0011] Croissant, Y. (2013). Mlogit: Multinomial logit model. R package version 0.2‐4. Retrieved, 13.

[ece33210-bib-0012] Daunt, F. , Afanasyev, V. , Adam, A. , Croxall, J. P. , & Wanless, S. (2007). From cradle to early grave: Juvenile mortality in European shags *Phalacrocorax aristotelis* results from inadequate development of foraging proficiency. Biology Letters, 3, 371–374. 17504733 10.1098/rsbl.2007.0157 PMC2390668

[ece33210-bib-0013] De Grissac, S. , Börger, L. , Guitteaud, A. , & Weimerskirch, H. (2016). Contrasting movement strategies among juvenile albatrosses and petrels. Scientific Reports, 6, 26103. 27189182 10.1038/srep26103 PMC4870643

[ece33210-bib-0014] Dias, M. P. , Granadeiro, J. P. , & Catry, P. (2012). Do seabirds differ from other migrants in their travel arrangements? On route strategies of cory's shearwater during its trans–equatorial journey. PLoS One, 7, e49376. 23145168 10.1371/journal.pone.0049376 PMC3492286

[ece33210-bib-0015] Dingemanse, N. J. , Both, C. , Drent, P. J. , van Oers, K. , & van Noordwijk, A. J. (2002). Repeatability and heritability of exploratory behaviour in great tits from the wild. Animal Behaviour, 64, 929–938.

[ece33210-bib-0016] Dingemanse, N. J. , Both, C. , van Noordwijk, A. J. , Rutten, A. L. , & Drent, P. J. (2003). Natal dispersal and personalities in great tits (*Parus major*). Proceedings of the Royal Society of London. Series B: Biological Sciences, 270, 741–747. 12713749 10.1098/rspb.2002.2300 PMC1691302

[ece33210-bib-0017] Fagan, W. F. , Cantrell, R. S. , Cosner, C. , Mueller, T. , & Noble, A. E. (2012). Leadership, social learning, and the maintenance (or collapse) of migratory populations. Theoretical Ecology, 5, 253–264.

[ece33210-bib-0018] Fauchald, P. (2009). Spatial interaction between seabirds and prey: Review and synthesis. Marine Ecology Progress Series, 391, 139–151.

[ece33210-bib-0019] Fauchald, P. , & Tveraa, T. (2003). Using first‐passage time in the analysis of area‐restricted search and habitat selection. Ecology, 84, 282–288.

[ece33210-bib-0020] Fay, R. , Barbraud, C. , Delord, K. , & Weimerskirch, H. (2016). Paternal but not maternal age influences early‐life performance of offspring in a long‐lived seabird. Proceedings of the Royal Society B: Biological Sciences, 283, 20152318. 27053738 10.1098/rspb.2015.2318 PMC4843644

[ece33210-bib-0021] Fay, R. , Weimerskirch, H. , Delord, K. , & Barbraud, C. (2015). Population density and climate shape early‐life survival and recruitment in a long‐lived pelagic seabird. Journal of Animal Ecology, 84, 1423–1433. 25976400 10.1111/1365-2656.12390

[ece33210-bib-0022] Felicísimo, Á. M. , Muñoz, J. , & González‐Solis, J. (2008). Ocean surface winds drive dynamics of transoceanic aerial movements. PLoS One, 3, e2928. 18698354 10.1371/journal.pone.0002928 PMC2491555

[ece33210-bib-0023] Fernandez‐Duque, E. (2003). Influences of moonlight, ambient temperature, and food availability on the diurnal and nocturnal activity of owl monkeys *(Aotus azarai*). Behavioral Ecology and Sociobiology, 54, 431–440.

[ece33210-bib-0024] Freeman, R. , Dean, B. , Kirk, H. , Leonard, K. , Phillips, R. A. , Perrins, C. M. , & Guilford, T. (2013). Predictive ethoinformatics reveals the complex migratory behaviour of a pelagic seabird, the Manx Shearwater. Journal of The Royal Society Interface, 10, 20130279. 10.1098/rsif.2013.0279 PMC3673166 23635496

[ece33210-bib-0025] Friedland, K. D. , Stock, C. , Drinkwater, K. F. , Link, J. S. , Leaf, R. T. , Shank, B. V. , … Fogarty, M. J. (2012). Pathways between primary production and fisheries yields of large marine ecosystems. PLoS One, 7, e28945. 22276100 10.1371/journal.pone.0028945 PMC3262787

[ece33210-bib-0026] Garriga, J. , Palmer, J. R. B. , Oltra, A. , & Bartumeus, F. (2016). Expectation–maximization binary clustering for behavioural annotation. PLoS One, 11, e0151984. 27002631 10.1371/journal.pone.0151984 PMC4803255

[ece33210-bib-0027] Hazen, E. , Maxwell, S. , Bailey, H. , Bograd, S. , Hamann, M. , Gaspar, P. , … Shillinger, G. (2012). Ontogeny in marine tagging and tracking science: Technologies and data gaps. Marine Ecology Progress Series, 457, 221–240.

[ece33210-bib-0028] Hijmans, R. J. (2015). Geosphere: Spherical trigonometry. R package version 1.5‐1.

[ece33210-bib-0029] Horswill, C. , Matthiopoulos, J. , Green, J. A. , Meredith, M. P. , Forcada, J. , Peat, H. , … Ratcliffe, N. (2014). Survival in macaroni penguins and the relative importance of different drivers: Individual traits, predation pressure and environmental variability. Journal of Animal Ecology, 83, 1057–1067. 24846695 10.1111/1365-2656.12229 PMC4284017

[ece33210-bib-0030] van den Hout, P. J. , van Gils, J. A. , Robin, F. , van der Geest, M. , Dekinga, A. , & Piersma, T. (2014). Interference from adults forces young red knots to forage for longer and in dangerous places. Animal Behaviour, 88, 137–146.

[ece33210-bib-0031] Hunt, G. L. (1991). Occurrence of polar seabirds at sea in relation to prey concentrations and oceanographic factors. Polar Research, 10, 553–560.

[ece33210-bib-0032] Hunt, G. L. , Mehlum, F. , Russell, R. W. , Irons, D. , Decker, M. B. , & Becker, P. H. (1999). Physical processes, prey abundance, and the foraging ecology of seabirds *Proceedings of the International Ornithological Congress* (pp. 2040–2056).

[ece33210-bib-0033] Hyrenbach, K. , Veit, R. , Weimerskirch, H. , & Hunt, L. Jr (2006). Seabird associations with mesoscale eddies: The subtropical Indian Ocean. Marine Ecology Progress Series, 324, 271–279.

[ece33210-bib-0034] Kai, E. T. , Rossi, V. , Sudre, J. , Weimerskirch, H. , Lopez, C. , Hernandez‐Garcia, E. , … Garçon, V. (2009). Top marine predators track Lagrangian coherent structures. Proceedings of the National Academy of Sciences of the United States of America, 106, 8245–8250. 19416811 10.1073/pnas.0811034106 PMC2677090

[ece33210-bib-0035] Kappes, M. (2009). Comparative foraging ecology and energetics of albatrosses. Santa Cruz: University of California.

[ece33210-bib-0036] Kareiva, P. , & Odell, G. (1987). Swarms of predators exhibit “preytaxis” if individual predators use area–restricted search. American Naturalist, 130, 233–270.

[ece33210-bib-0037] Krebs, J. R. (1982). Territorial defence in the great tit (*Parus major*): Do residents always win? Behavioral Ecology and Sociobiology, 11, 185–194.

[ece33210-bib-0038] Lazaridis, E. (2014). Lunar: Lunar phase & distance, seasons and other environmental factors. R package version 0.1‐04.

[ece33210-bib-0039] Lewison, R. , Oro, D. , Godley, B. J. , Underhill, L. , Bearhop, S. , Wilson, R. P. , … Yorio, P. (2012). Research priorities for seabirds: Improving conservation and management in the 21st century. Endangered Species Research, 17, 93–121.

[ece33210-bib-0040] Lindström, J. (1999). Early development and fitness in birds and mammals. Trends in Ecology & Evolution, 14, 343–348. 10441307 10.1016/s0169-5347(99)01639-0

[ece33210-bib-0041] Louzao, M. , Pinaud, D. , Peron, C. , Delord, K. , Wiegand, T. , & Weimerskirch, H. (2011). Conserving pelagic habitats: Seascape modelling of an oceanic top predator. Journal of Applied Ecology, 48, 121–132.

[ece33210-bib-0042] Louzao, M. , Weigand, T. , Bartumeus, F. , & Weimerskirch, H. (2014). Coupling instantaneous energy–budget models and behavioural mode analysis to estimate optimal foraging strategy: An example with wandering albatrosses. Movement Ecology, 2, 8. 25520818 10.1186/2051-3933-2-8 PMC4267543

[ece33210-bib-0043] Mackley, E. K. , Phillips, R. A. , Silk, J. R. , Wakefield, E. D. , Afanasyev, V. , Fox, J. W. , & Furness, R. W. (2010). Free as a bird? Activity patterns of albatrosses during the nonbreeding period. Marine Ecology Progress Series, 406, 291–303.

[ece33210-bib-0044] Mackley, E. K. , Phillips, R. A. , Silk, J. R. D. , Wakefield, E. D. , Afanasyev, V. , & Furness, R. W. (2010). At‐sea activity patterns of breeding and nonbreeding white‐chinned petrels *Procellaria aequinoctialis* from South Georgia. Marine Biology, 158, 429–438.

[ece33210-bib-0045] Magrath, R. D. (1991). Nestling weight and juvenile survival in the blackbird, *Turdus merula* . The Journal of Animal Ecology, 60, 335–351.

[ece33210-bib-0046] Marchetti, K. , & Price, T. (1989). Differences in the foraging of juvenile and adult birds: The importance of developmental constraints. Biological Reviews, 64, 51–70.

[ece33210-bib-0047] McGillicuddy, D. J. , Anderson, L. A. , Bates, N. R. , Bibby, T. , Buesseler, K. O. , Carlson, C. A. , … Steinberg, D. K. (2007). Eddy/wind interactions stimulate extraordinary mid‐ocean plankton blooms. Science, 316, 1021–1026. 17510363 10.1126/science.1136256

[ece33210-bib-0048] Mueller, T. , O'Hara, R. B. , Converse, S. J. , Urbanek, R. P. , & Fagan, W. F. (2013). Social learning of migratory performance. Science, 341, 999–1002. 23990559 10.1126/science.1237139

[ece33210-bib-0049] Naef‐Daenzer, B. , Widmer, F. , & Nuber, M. (2001). Differential post‐fledging survival of great and coal tits in relation to their condition and fledging date. Journal of Animal Ecology, 70, 730–738.

[ece33210-bib-0050] Nevitt, G. A. (2000). Olfactory foraging by Antarctic procellariiform seabirds: Life at high Reynolds numbers. The Biological Bulletin, 198, 245–253. 10786944 10.2307/1542527

[ece33210-bib-0051] Nevoux, M. , Weimerskirch, H. , & Barbraud, C. (2007). Environmental variation and experience‐related differences in the demography of the long‐lived black‐browed albatross. Journal of Animal Ecology, 76, 159–167. 17184364 10.1111/j.1365-2656.2006.01191.x

[ece33210-bib-0052] Newton, I. (2008). The migration ecology of birds. Academic Press, Oxford, UK.

[ece33210-bib-0053] Nicholls, D. , Robertson, C. , Prince, P. , Murray, M. , Walker, K. , & Elliott, G. (2002). Foraging niches of three Diomedea albatrosses. Marine Ecology Progress Series, 231, 269–277.

[ece33210-bib-0054] Paiva, V. H. , Geraldes, P. , Ramírez, I. , Garthe, S. , & Ramos, J. A. (2010). How area restricted search of a pelagic seabird changes while performing a dual foraging strategy. Oikos, 119, 1423–1434.

[ece33210-bib-0055] Patrick, S. C. , & Weimerskirch, H. (2015). Senescence rates and late adulthood reproductive success are strongly influenced by personality in a long‐lived seabird. Proceedings of the Royal Society B: Biological Sciences, 282, 20141649. 25473008 10.1098/rspb.2014.1649 PMC4286031

[ece33210-bib-0056] Pennycuick, C. J. (1982). The flight of petrels and albatrosses (Procellariiformes), observed in South Georgia and its vicinity. Philosophical Transactions of the Royal Society of London B, Biological Sciences, 300, 75–106.

[ece33210-bib-0057] Perrin, M. R. (1979). The roles of reproduction, survival, and territoriality in the seasonal dynamics of Clethrionomys gapperi populations. Acta Theriologica, 24, 475–500.

[ece33210-bib-0058] Phillips, R. A. , Xavier, J. C. , Croxall, J. P. , & Burger, A. E. (2003). Effects of satellite transmitters on albatrosses and petrels. The Auk, 120, 1082–1090.

[ece33210-bib-0059] Pinaud, D. , & Weimerskirch, H. (2007). At‐sea distribution and scale‐dependent foraging behaviour of petrels and albatrosses: A comparative study. Journal of Animal Ecology, 76, 9–19. 17184348 10.1111/j.1365-2656.2006.01186.x

[ece33210-bib-0060] Pinet, P. , Jaeger, A. , Cordier, E. , Potin, G. , & Le Corre, M. (2011). Celestial moderation of tropical seabird behavior. PLoS One, 6, e27663. 22110711 10.1371/journal.pone.0027663 PMC3215727

[ece33210-bib-0061] R Core Team . (2015). R: A language and environment for statistical computing. Vienna, Austria: R Foundation for Statistical Computing.

[ece33210-bib-0062] Regular, P. M. , Hedd, A. , & Montevecchi, W. A. (2011). Fishing in the dark: A pursuit‐diving seabird modifies foraging behaviour in response to nocturnal light levels. PLoS One, 6, e26763. 22046348 10.1371/journal.pone.0026763 PMC3202575

[ece33210-bib-0063] Riotte‐Lambert, L. , & Weimerskirch, H. (2013). Do naive juvenile seabirds forage differently from adults? Proceedings of the Royal Society B: Biological Sciences, 280, 20131434. 23926153 10.1098/rspb.2013.1434 PMC3757974

[ece33210-bib-0064] Salamolard, M. , & Weimerskirch, H. (1993). Relationship between foraging effort and energy requirement throughout the breeding season in the wandering albatross. Functional Ecology, 7, 643.

[ece33210-bib-0065] Sandell, M. , & Smith, H. G. (1991). Dominance, prior occupancy, and winter residency in the great tit (parus major). Behavioral Ecology and Sociobiology, 29, 147–152.

[ece33210-bib-0066] Scales, K. L. , Miller, P. I. , Hawkes, L. A. , Ingram, S. N. , Sims, D. W. , & Votier, S. C. (2014). Review: On the Front Line: Frontal zones as priority at‐sea conservation areas for mobile marine vertebrates (ed A Punt). Journal of Applied Ecology, 51, 1575–1583.

[ece33210-bib-0067] Scales, K. L. , Miller, P. I. , Ingram, S. N. , Hazen, E. L. , Bograd, S. J. , & Phillips, R. A. (2016). Identifying predictable foraging habitats for a wide‐ranging marine predator using ensemble ecological niche models. Diversity and Distributions, 22, 212–224.

[ece33210-bib-0068] Sergio, F. , Schmitz, O. J. , Krebs, C. J. , Holt, R. D. , Heithaus, M. R. , Wirsing, A. J. , … Korpimäki, E. (2014). Towards a cohesive, holistic view of top predation: A definition, synthesis and perspective. Oikos, 123, 1234–1243.

[ece33210-bib-0069] Shaffer, S. A. , Costa, D. P. , & Weimerskirch, H. (2001). Behavioural factors affecting foraging effort of breeding wandering albatrosses. Journal of Animal Ecology, 70, 864–874.

[ece33210-bib-0070] Sol, D. , Santos, D. M. , Garcia, J. , & Cuadrado, M. (1998). Competition for food in urban pigeons: The cost of being juvenile. The Condor, 100, 298–304.

[ece33210-bib-0071] Soutullo, A. , Urios, V. , Ferrer, M. , & Peñarrubia, S. G. (2006). Post‐fledging behaviour in Golden Eagles *Aquila chrysaetos*: Onset of juvenile dispersal and progressive distancing from the nest. Ibis, 148, 307–312.

[ece33210-bib-0072] Stamps, J. A. (1987). The effect of familiarity with a neighborhood on territory acquisition. Behavioral Ecology and Sociobiology, 21, 273–277.

[ece33210-bib-0073] Thorup, K. , Alerstam, T. , Hake, M. , & Kjellén, N. (2003). Bird orientation: Compensation for wind drift in migrating raptors is age dependent. Proceedings of the Royal Society of London. Series B: Biological Sciences, 270, S8–S11. 12952622 10.1098/rsbl.2003.0014 PMC1698035

[ece33210-bib-0074] Tosh, C. A. , de Bruyn, P. J. N. , Steyn, J. , Bornemann, H. , van den Hoff, J. , Stewart, B. S. , … Bester, M. N. (2015). The importance of seasonal sea surface height anomalies for foraging juvenile southern elephant seals. Marine Biology, 162, 2131–2140.

[ece33210-bib-0075] Verbeek, M. E. M. , Drent, P. J. , & Wiepkema, P. R. (1994). Consistent individual differences in early exploratory behaviour of male great tits. Animal Behaviour, 48, 1113–1121.

[ece33210-bib-0076] Waugh, S. M. , & Weimerskirch, H. (2003). Environmental heterogeneity and the evolution of foraging behaviour in long ranging greater albatrosses. Oikos, 103, 374–384.

[ece33210-bib-0077] Weimerskirch, H. (1992). Reproductive effort in long‐lived birds: Age‐specific patterns of condition, reproduction and survival in the wandering albatross. Oikos, 64, 464.

[ece33210-bib-0078] Weimerskirch, H. (2007). Are seabirds foraging for unpredictable resources? Deep Sea Research Part II: Topical Studies in Oceanography, 54, 211–223.

[ece33210-bib-0079] Weimerskirch, H. , Akesson, S. , & Pinaud, D. (2006). Postnatal dispersal of wandering albatrosses *Diomedea exulans*: Implications for the conservation of the species. Journal of Avian Biology, 37, 23–28.

[ece33210-bib-0080] Weimerskirch, H. , Barbraud, C. , & Lys, P. (2000). Sex differences in parental investment and chick growth in wandering albatrosses: Fitness consequences. Ecology, 81, 309–318.

[ece33210-bib-0081] Weimerskirch, H. , Bonadonna, F. , Bailleul, F. , Mabille, G. , Dell'Omo, G. , & Lipp, H.‐P. (2002). GPS tracking of foraging albatrosses. Science, 295, 1259. 11847332 10.1126/science.1068034

[ece33210-bib-0082] Weimerskirch, H. , Cherel, Y. , Cuenot‐Chaillet, F. , & Ridoux, V. (1997). Alternative foraging strategies and resource allocation by male and female wandering albatrosses. Ecology, 78, 2051–2063.

[ece33210-bib-0083] Weimerskirch, H. , Delord, K. , Guitteaud, A. , Phillips, R. A. , & Pinet, P. (2015). Extreme variation in migration strategies between and within wandering albatross populations during their sabbatical year, and their fitness consequences. Scientific Reports, 5, 8853. 25747757 10.1038/srep08853 PMC4352845

[ece33210-bib-0084] Weimerskirch, H. , Gault, A. , & Cherel, Y. (2005). Prey distribution and patchiness: Factors in foraging success and efficiency of wandering albatrosses. Ecology, 86, 2611–2622.

[ece33210-bib-0085] Weimerskirch, H. , Guionnet, T. , Martin, J. , Shaffer, S. A. , & Costa, D. P. (2000). Fast and fuel efficient? Optimal use of wind by flying albatrosses. Proceedings of the Royal Society of London. Series B: Biological Sciences, 267, 1869–1874. 11052538 10.1098/rspb.2000.1223 PMC1690761

[ece33210-bib-0086] Weimerskirch, H. , Lallemand, J. , & Martin, J. (2005). Population sex ratio variation in a monogamous long‐lived bird, the wandering albatross. Journal of Animal Ecology, 74, 285–291.

[ece33210-bib-0087] Weimerskirch, H. , Pinaud, D. , Pawlowski, F. , & Bost, C.‐A. (2007). Does prey capture induce area‐restricted search? A fine‐scale study using gps in a marine predator, the wandering albatross. The American Naturalist, 170, 734–743. 10.1086/522059 17926295

[ece33210-bib-0088] Wheelwright, N. T. , & Templeton, J. J. (2003). Development of foraging skills and the transition to independence in juvenile savannah sparrows. The Condor, 105, 279–287.

[ece33210-bib-0089] Wilson, A. J. , & Nussey, D. H. (2010). What is individual quality? An evolutionary perspective. Trends in Ecology & Evolution, 25, 207–214. 19897275 10.1016/j.tree.2009.10.002

[ece33210-bib-0090] Wunderle, J. M. (1991). Age‐specific foraging proficiency in birds. Current Ornithology, 8, 273–324.

[ece33210-bib-0091] Wunderle, J. M. Jr , & Martinez, J. S. (1987). Spatial learning in the nectarivorous bananaquit: Juveniles versus adults. Animal Behaviour, 35, 652–658.

[ece33210-bib-0092] Yamamoto, T. , Takahashi, A. , Yoda, K. , Katsumata, N. , Watanabe, S. , Sato, K. , & Trathan, P. N. (2008). The lunar cycle affects at‐sea behaviour in a pelagic seabird, the streaked shearwater, *Calonectris leucomelas* . Animal Behaviour, 76, 1647–1652.

[ece33210-bib-0093] Yoda, K. , Kohno, H. , & Naito, Y. (2004). Development of flight performance in the brown booby. Proceedings of the Royal Society of London B: Biological Sciences, 271, S240–S242. 10.1098/rsbl.2003.0157 PMC1810025 15252995

