# Supplementary material for: Early‐life foraging: Behavioral responses of newly fledged albatrosses to environmental conditions
Source: Ecol Evol. 2017 Jul 26;7(17):6766–78. doi: 10.1002/ece3.3210 (PMC5587467; doi:10.1002/ece3.3210)
Supplement: Supplementary file 1 [file ECE3-7-6766-s001.docx]

**Early life foraging: behavioural responses of newly fledged albatrosses to environmental conditions**

Sophie de Grissac , Frederic Bartumeus, Samantha L. Cox, Henri Weimerskirch

# Supplementary Information

### Table S1. Statistics of the behavioural modes obtained with the EMbC algorithm.

|  |  | | **RESTING** | | | **ACTIVE SITTING** | | | **BALLISTIC** | | | **DIFFUSIVE** | | |
| --- | --- | --- | --- | --- | --- | --- | --- | --- | --- | --- | --- | --- | --- | --- |
| **Velocity** (km.h^-1^) | | mean ± sd | 1.27 | ± | 0.68 | 0.94 | ± | 0.55 | 28.08 | ± | 15.50 | 17.82 | ± | 12.68 |
|  |  | min - max | 0.03 | - | 3.43 | 0.01 | - | 2.34 | 3.29 | - | 80.99 | 2.33 | - | 72.82 |
| **Turning angle** (rad) | | mean ± sd | 0.27 | ± | 0.19 | 1.57 | ± | 0.70 | 0.21 | ± | 0.15 | 1.45 | ± | 0.74 |
|  |  | min - max | 0.00 | - | 0.77 | 0.68 | - | 3.14 | 0.00 | - | 0.65 | 0.51 | - | 3.14 |

### Fig.S1. Histograms showing proportions of each behaviour by day (top) and night (bottom) of juveniles (left) and adults (right), all positions included (yellow: resting; red: active sitting; blue: ballistic movement; light blue: diffusive movement).


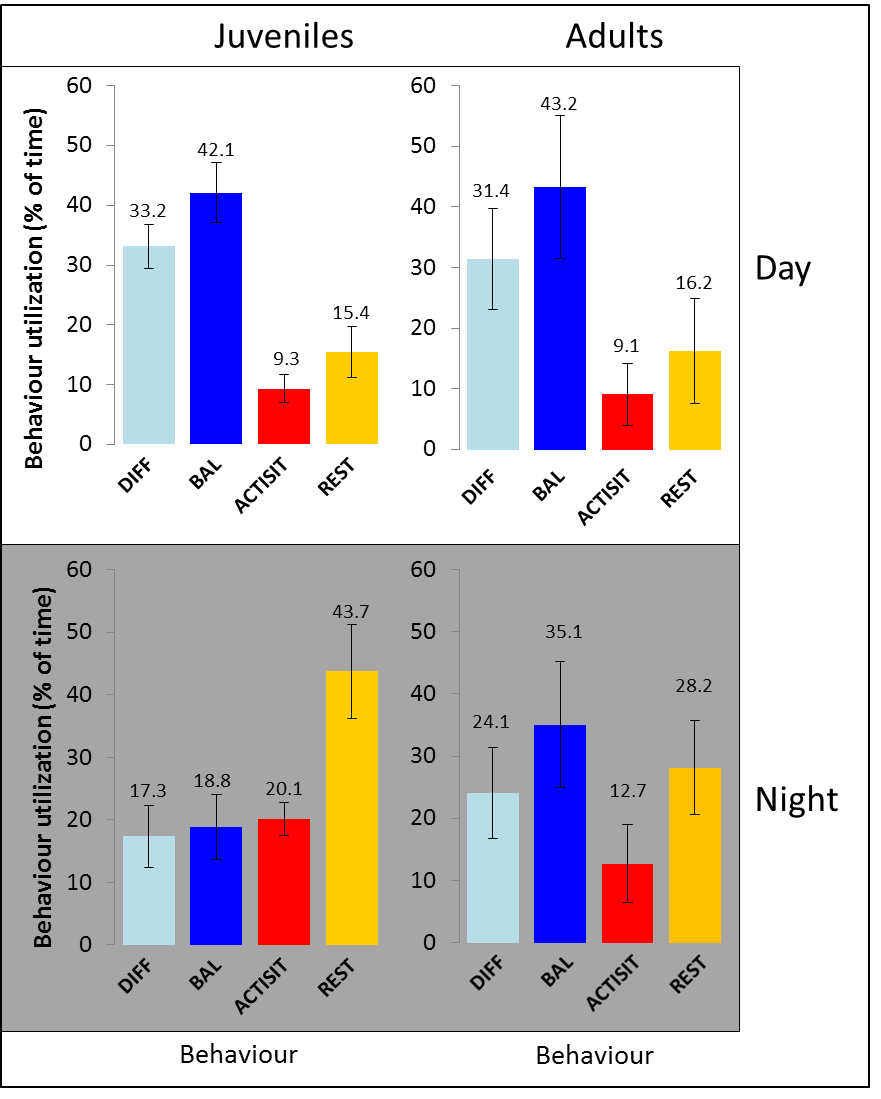


### Fig.S2. Portions of juvenile’s trajectories following the continental shelf break.


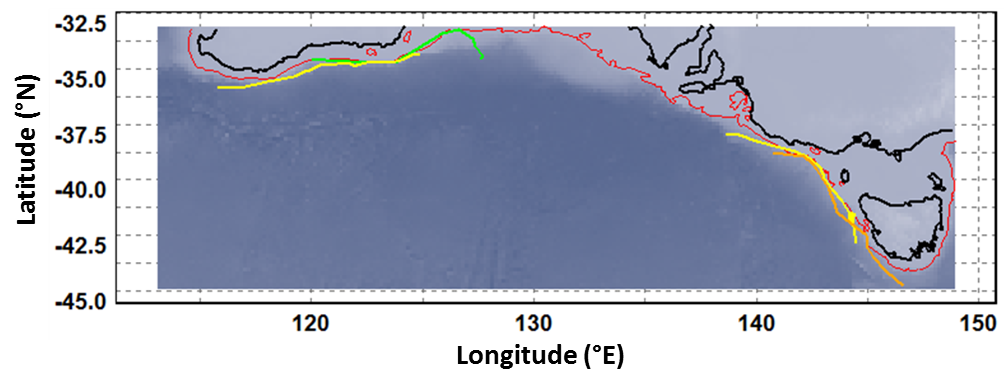


The coast line is in black and the red lines are -200m isobaths whereas the blue gradient represents the bathymetry. The four segments correspond to the behaviour of 3 different juveniles, two females (yellow and green) and one male (orange), last 48 hours (approximately 21 consecutive GPS locations) and occur at different time of years (March, August, October).

### Fig.S3. Variability in the bathymetric characteristics of habitats visited by individual juveniles during their trips.


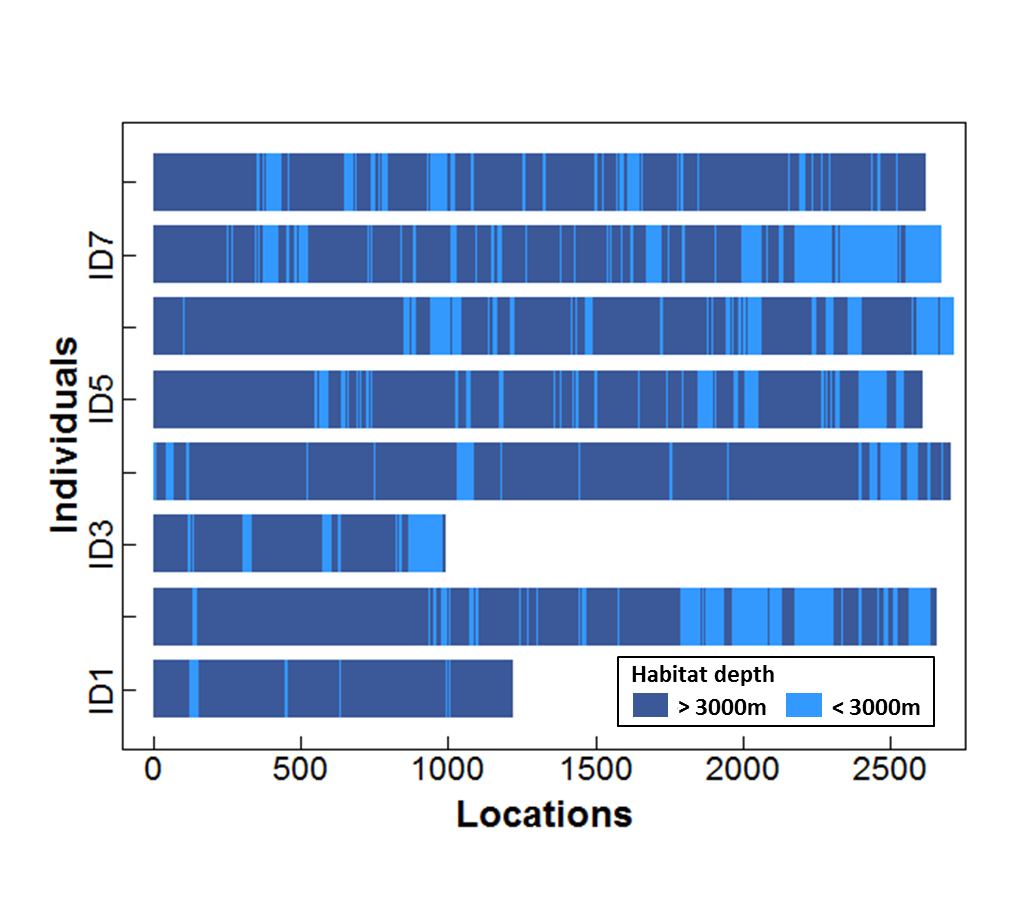


Each GPS location was assigned a color according to corresponding bathymetry: pelagic (dark blue is deeper than 3000m) or over continental slopes or other oceanic features (light blue, above 3000m).

**Fig.S4. Proportion of behavioural modes used by juvenile males and females at the beginning of their trips (A) and later (B).** Differences are significant only for the resting mode (LL) and ballistic mode (HL) and during the first 3 months (stars indicate linear mixed model p<0.05).

**< 3 months**

**> 3 months**

******

*
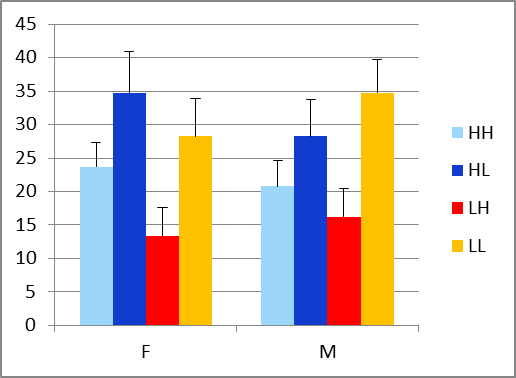

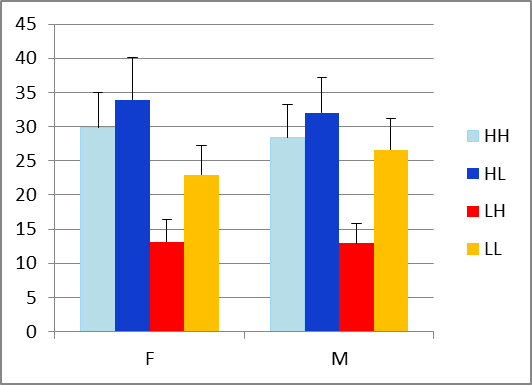
*

******

### **Fig.S5. Predicted probabilities for juveniles and adults to use each of the four behaviours at night according to moonlight intensity.** Dashed lines are 95% IC.

**
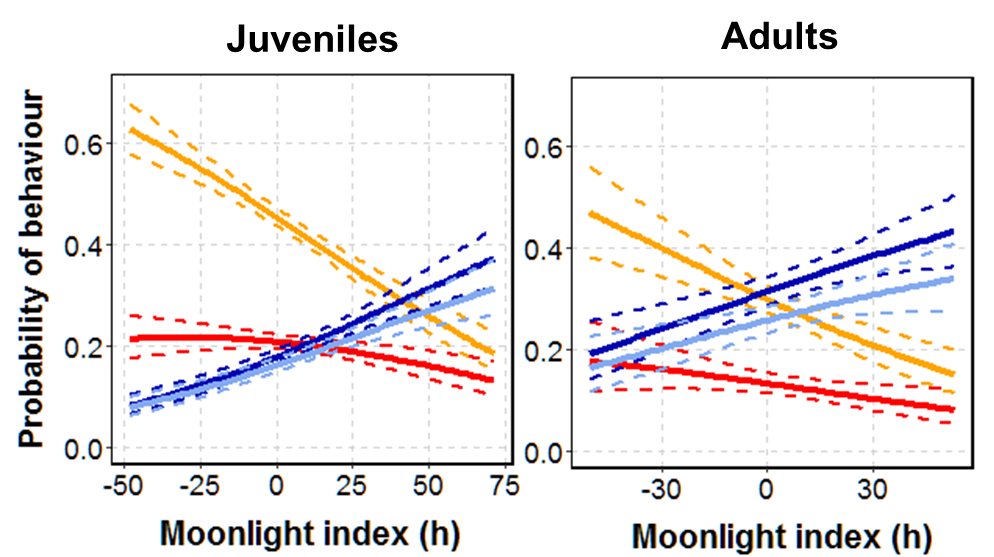
**

### Table S2. Summary of the four multinomial logistic regression model outputs. A) Juvenile diurnal behaviour, B) juvenile nocturnal behaviour, C) adult diurnal behaviour, D) adult nocturnal behaviour. Each table correspond to the testing of one response variable included in the model. Numbers are estimated effect of that variable on the probability to perform a behaviour (named tested behaviour) in reference to another (reference behaviour). Each combination of two behaviours has been tested by changing the reference behaviour in the model. Significant effects (P < 0.05) are in bold.

| **SI-2.A. JUVENILES – DIURNAL BEHAVIOUR** | | | | |
| --- | --- | --- | --- | --- |
|  |  |  |  |  |
| **Reference behaviour** | **Tested Behaviour** | | | |
| Intercept | Resting | Active sitting | Balistic | Diffusive |
| Resting |  | **-0.47** | **1.16** | **0.97** |
| Active sitting |  |  | **1.63** | **1.43** |
| Balistic |  |  |  | **-0.19** |
| Diffusive |  |  |  |  |
|  |  |  |  |  |
|  |  |  |  |  |
| Wind speed | Resting | Active sitting | Balistic | Diffusive |
| Resting |  | **-0.18** | **0.37** | **0.29** |
| Active sitting |  |  | **0.55** | **0.48** |
| Balistic |  |  |  | -0.06 |
| Diffusive |  |  |  |  |
|  |  |  |  |  |
|  |  |  |  |  |
| Bathymetry | Resting | Active sitting | Balistic | Diffusive |
| Resting |  | 0.06 | **-0.15** | **0.14** |
| Active sitting |  |  | **-0.21** | 0.08 |
| Balistic |  |  |  | **0.29** |
| Diffusive |  |  |  |  |
|  |  |  |  |  |
|  |  |  |  |  |
| log(CHLa) | Resting | Active sitting | Balistic | Diffusive |
| Resting |  | **0.19** | **0.34** | **0.3** |
| Active sitting |  |  | **0.15** | -0.11 |
| Balistic |  |  |  | 0.04 |
| Diffusive |  |  |  |  |

| **SI.2.B. JUVENILES – NOCTURNAL BEHAVIOUR** | | | | |
| --- | --- | --- | --- | --- |
|  |  |  |  |  |
| **Reference behaviour** | **Tested Behaviour** | | | |
| Intercept | Resting | Active sitting | Balistic | Diffusive |
| Resting |  | **-0.86** | **-1.14** | **-1.32** |
| Active sitting |  |  | **-0.28** | **-0.46** |
| Balistic |  |  |  | **-0.18** |
| Diffusive |  |  |  |  |
|  |  |  |  |  |
|  |  |  |  |  |
| Wind speed | Resting | Active sitting | Balistic | Diffusive |
| Resting |  | -0.019 | **0.4** | **0.28** |
| Active sitting |  |  | **0.42** | **0.3** |
| Balistic |  |  |  | **-0.11** |
| Diffusive |  |  |  |  |
|  |  |  |  |  |
|  |  |  |  |  |
| Bathymetry | Resting | Active sitting | Balistic | Diffusive |
| Resting |  | 0.07 | **-0.2** | **0.14** |
| Active sitting |  |  | **-0.27** | 0.07 |
| Balistic |  |  |  | **0.35** |
| Diffusive |  |  |  |  |
|  |  |  |  |  |
|  |  |  |  |  |
| Night length | Resting | Active sitting | Balistic | Diffusive |
| Resting |  | 0.05 | **0.4** | **0.48** |
| Active sitting |  |  | **0.36** | **0.44** |
| Balistic |  |  |  | 0.08 |
| Diffusive |  |  |  |  |
|  |  |  |  |  |
| Moonlight | Resting | Active sitting | Balistic | Diffusive |
| Resting |  | **0.12** | **0.46** | **0.44** |
| Active sitting |  |  | **0.34** | **0.32** |
| Balistic |  |  |  | -0.02 |
| Diffusive |  |  |  |  |

| **SI.2.C. ADULTS – DIURNAL BEHAVIOUR** | | | | |
| --- | --- | --- | --- | --- |
|  |  |  |  |  |
| **Reference behaviour** | **Tested Behaviour** | | | |
| Intercept | Resting | Active sitting | Balistic | Diffusive |
| Resting |  | **-0.71** | **0.68** | **0.42** |
| Active sitting |  |  | **1.46** | **1.2** |
| Balistic |  |  |  | **-0.27** |
| Diffusive |  |  |  |  |
|  |  |  |  |  |
|  |  |  |  |  |
| Wind speed | Resting | Active sitting | Balistic | Diffusive |
| Resting |  | **0.14** | **0.4** | **0.37** |
| Active sitting |  |  | **0.27** | **0.26** |
| Balistic |  |  |  | -0.01 |
| Diffusive |  |  |  |  |
|  |  |  |  |  |
|  |  |  |  |  |
| Bathymetry | Resting | Active sitting | Balistic | Diffusive |
| Resting |  | 0.12 | **-0.27** | **0.11** |
| Active sitting |  |  | **-0.38** | 0.04 |
| Balistic |  |  |  | **0.42** |
| Diffusive |  |  |  |  |
|  |  |  |  |  |
|  |  |  |  |  |
| SLA | Resting | Active sitting | Balistic | Diffusive |
| Resting |  | -0.12 | **-0.24** | **-0.23** |
| Active sitting |  |  | -0.11 | -0.07 |
| Balistic |  |  |  | 0.05 |
| Diffusive |  |  |  |  |

| **SI.2.D. ADULTS – NOCTURNAL BEHAVIOUR** | | | | |
| --- | --- | --- | --- | --- |
|  |  |  |  |  |
| **Reference behaviour** | **Tested Behaviour** | | | |
| Intercept | Resting | Active sitting | Balistic | Diffusive |
| Resting |  | **-0.79** | **0.17** | -0.03 |
| Active sitting |  |  | **0.96** | **0.84** |
| Balistic |  |  |  | **-0.2** |
| Diffusive |  |  |  |  |
|  |  |  |  |  |
|  |  |  |  |  |
| Wind speed | Resting | Active sitting | Balistic | Diffusive |
| Resting |  | **0.16** | **0.4** | **0.36** |
| Active sitting |  |  | **0.96** | **0.36** |
| Balistic |  |  |  | -0.03 |
| Diffusive |  |  |  |  |
|  |  |  |  |  |
|  |  |  |  |  |
| Bathymetry | Resting | Active sitting | Balistic | Diffusive |
| Resting |  | 0.01 | **-0.35** | **-0.2** |
| Active sitting |  |  | **-0.4** | **-0.29** |
| Balistic |  |  |  | **0.15** |
| Diffusive |  |  |  |  |
|  |  |  |  |  |
|  |  |  |  |  |
| SLA | Resting | Active sitting | Balistic | Diffusive |
| Resting |  | -0.12 | **-0.26** | **-0.2** |
| Active sitting |  |  | -0.19 | -0.02 |
| Balistic |  |  |  | 0.17 |
| Diffusive |  |  |  |  |

| Moon | Resting | Active sitting | Balistic | Diffusive |
| --- | --- | --- | --- | --- |
| Resting |  | 0.08 | **0.41** | **0.38** |
| Active sitting |  |  | **0.24** | **0.29** |
| Balistic |  |  |  | -0.03 |
| Diffusive |  |  |  |  |

**Figure S6. Autocorrelogram of the residuals of the multinomial logistic regression model « Juvenile diurnal behaviour » after removing positions at lag 1 to reduce autocorrelation of the residuals.** Plot has been computed with the R function acf(). Figure is split on 2 pages.


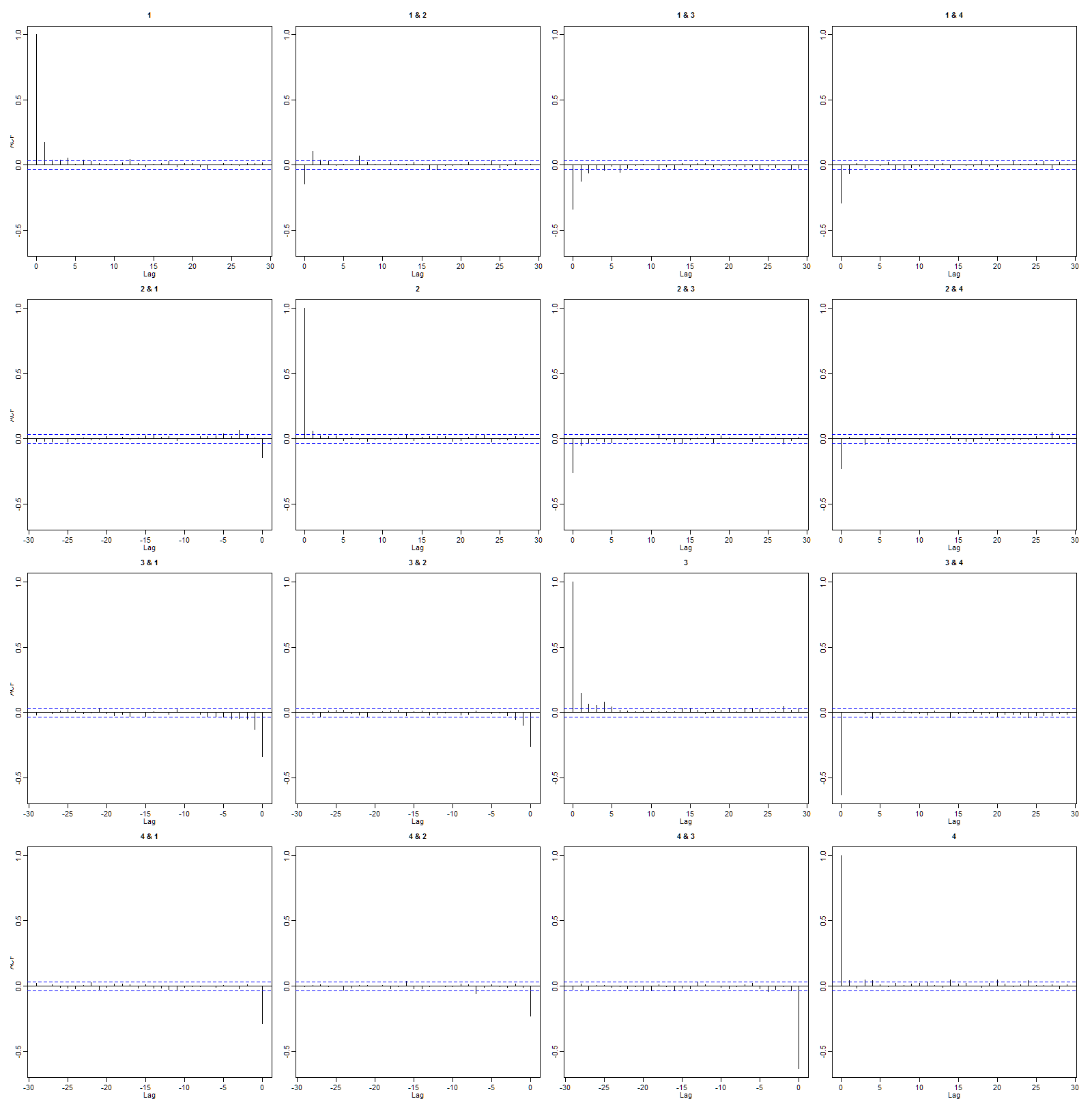

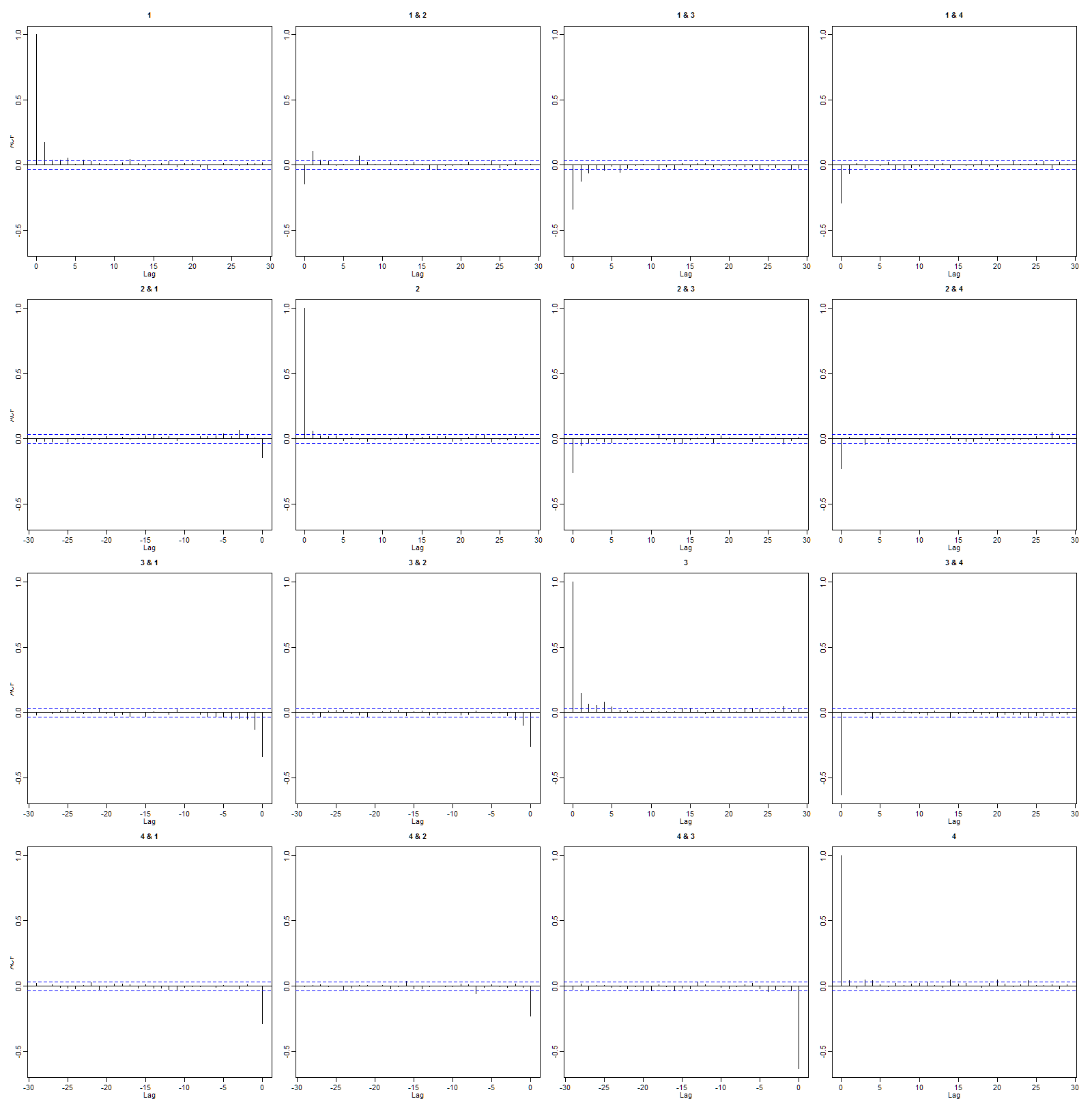


**Figure S7. Autocorrelogram of the residuals of the multinomial logistic regression model « Adult diurnal behaviour ». Sample size is not large enough to get rid of autocorrelation of max. lag 3.** Plot has been computed with the R function acf(). Figure is split on 2 pages.


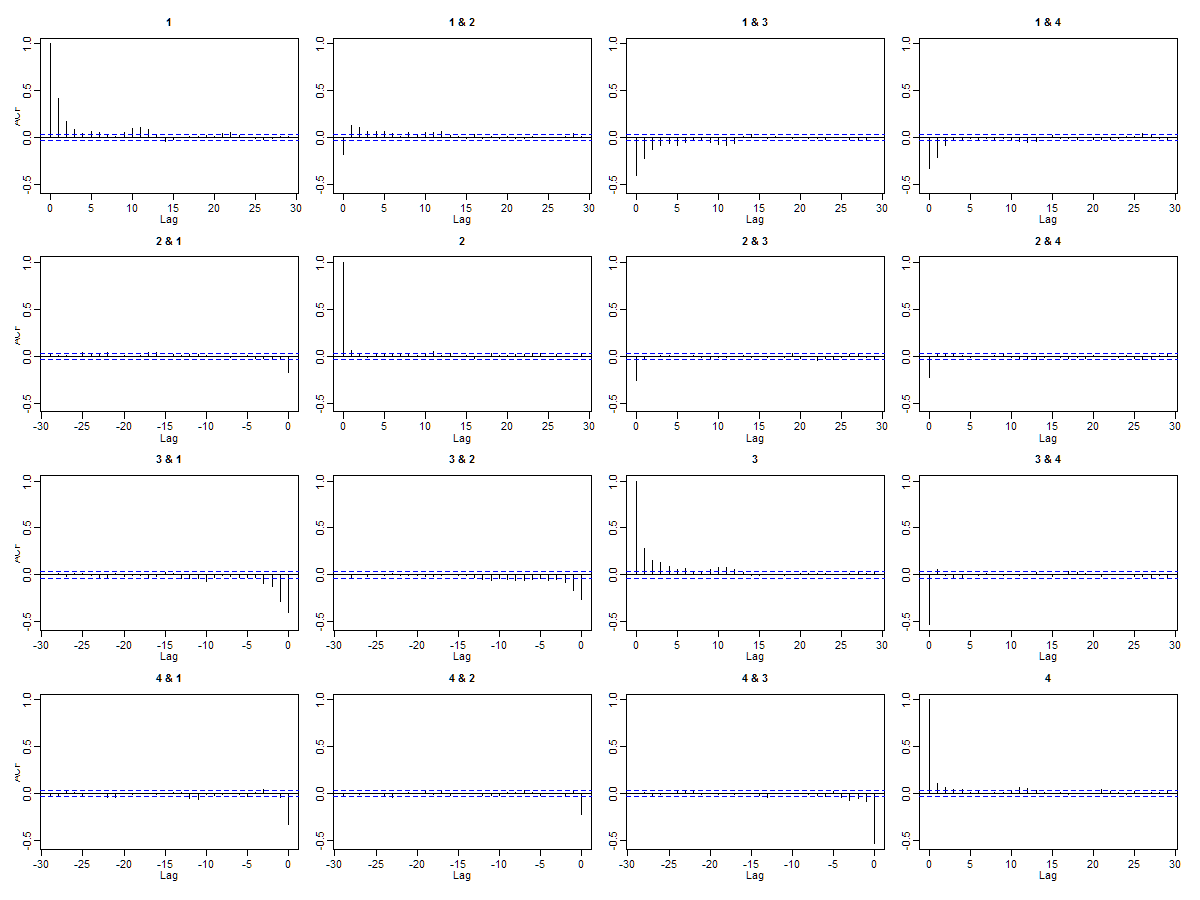


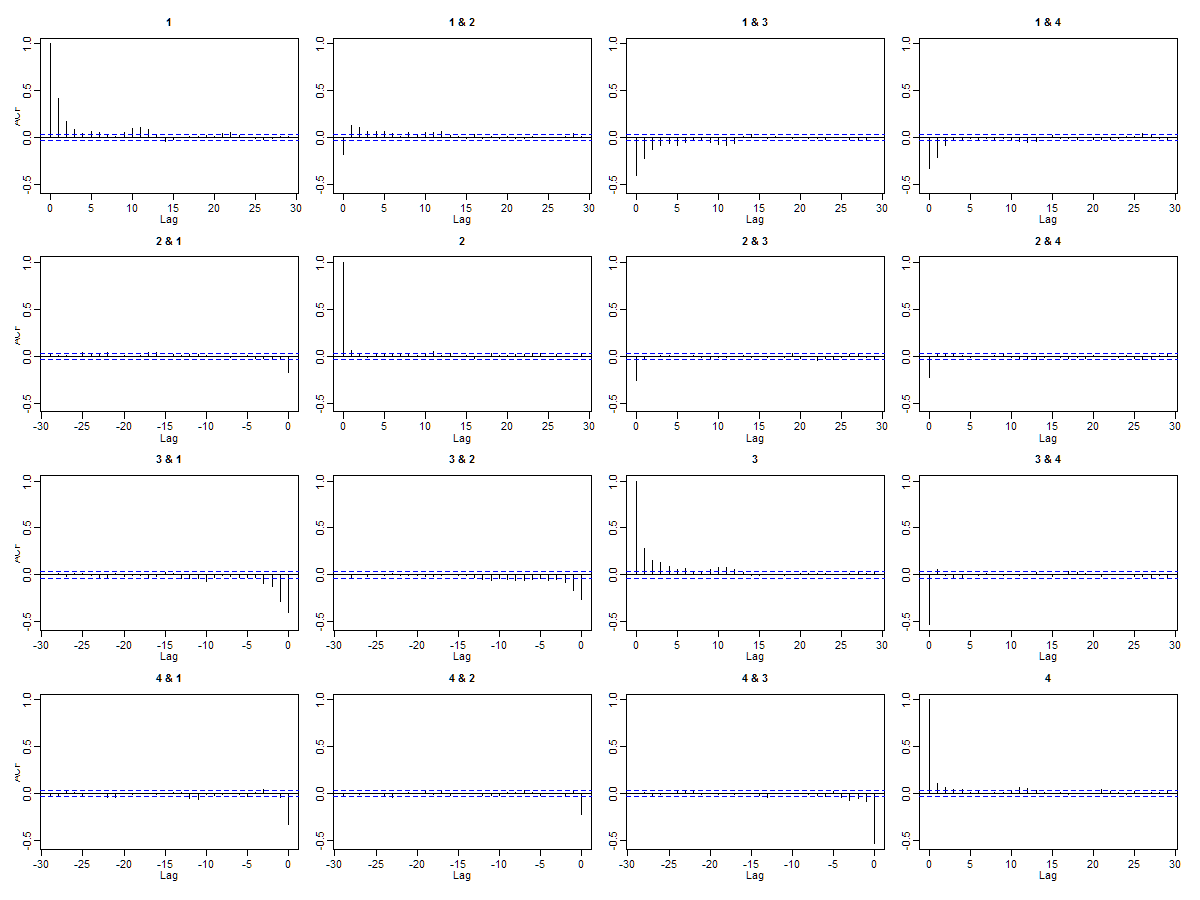


**Table S3. Outputs of multinomial logistic regressions performed on juvenile behaviour during day (1.a) and night (1.b) and for adult behaviour during day (2.a) and night (2.b).** Only models using the behaviour ‘Resting’ as reference behaviour (i.e. intercept, ‘reflevel = 1’) are shown here. Coefficients and significance tests of models with other behaviours as reference are summed up in the main body of the article (Table 2).

**Table SI-3.1.a. Juvenile diurnal behaviour**

Call:

mlogit(formula = lbl ~ 1 | scale(Swind) + scale(bathy) + scale(log(chla)),

data = JUV_night, reflevel = 1, method = "nr", print.level = 0)

Frequencies of alternatives:

1 2 3 4

0.139515 0.089255 0.424321 0.346909

nr method

5 iterations, 0h:0m:0s

g'(-H)^-1g = 0.00164

successive function values within tolerance limits

Coefficients :

Estimate Std. Error t-value Pr(>|t|)

2:(intercept) -0.462738 0.079100 -5.8500 4.916e-09 ***

3:(intercept) 1.168624 0.055649 21.0001 < 2.2e-16 ***

4:(intercept) 0.971561 0.056972 17.0532 < 2.2e-16 ***

2:scale(Swind) -0.180362 0.082557 -2.1847 0.028911 *

3:scale(Swind) 0.365547 0.058124 6.2891 3.194e-10 ***

4:scale(Swind) 0.298004 0.059308 5.0246 5.044e-07 ***

2:scale(bathy) 0.059585 0.075465 0.7896 0.429783

3:scale(bathy) -0.154122 0.057193 -2.6948 0.007043 **

4:scale(bathy) 0.143520 0.056562 2.5374 0.011169 *

2:scale(log(chla)) 0.189247 0.074427 2.5427 0.010999 *

3:scale(log(chla)) 0.341182 0.054942 6.2099 5.301e-10 ***

4:scale(log(chla)) 0.300593 0.056501 5.3201 1.037e-07 ***

---

Signif. codes: 0 ‘***’ 0.001 ‘**’ 0.01 ‘*’ 0.05 ‘.’ 0.1 ‘ ’ 1

MOST IMPORTANT VARIABLES (absolute value of the t-statistic)

**variables Overall**

scale(Swind) 0.8440112

scale(log(chla)) 0.8311112

scale(bathy) 0.3572670

**Table SI-3.1.b. Juvenile nocturnal behaviour**

Call:

mlogit(formula = lbl ~ 1 | scale(Swind) + scale(bathy) + scale(nightlength) + moon, data = JUV_night, reflevel = 1, method = "nr", print.level = 0)

Frequencies of alternatives:

1 2 3 4

0.41848 0.19547 0.20393 0.18212

nr method

5 iterations, 0h:0m:0s

g'(-H)^-1g = 2.26E-05

successive function values within tolerance limits

Coefficients :

Estimate Std. Error t-value Pr(>|t|)

2:(intercept) -0.740797 0.046867 -15.8064 < 2.2e-16 ***

3:(intercept) -0.775779 0.048275 -16.0701 < 2.2e-16 ***

4:(intercept) -0.880235 0.050121 -17.5623 < 2.2e-16 ***

2:scale(Swind) -0.020660 0.049887 -0.4141 0.678774

3:scale(Swind) 0.397227 0.049266 8.0629 6.661e-16 ***

4:scale(Swind) 0.278029 0.050224 5.5358 3.098e-08 ***

2:scale(bathy) 0.073709 0.047040 1.5669 0.117130

3:scale(bathy) -0.198934 0.050118 -3.9693 7.207e-05 ***

4:scale(bathy) 0.142038 0.047071 3.0175 0.002549 **

2:scale(nightlength) 0.036988 0.047894 0.7723 0.439947

3:scale(nightlength) 0.351722 0.051115 6.8811 5.941e-12 ***

4:scale(nightlength) 0.424446 0.054980 7.7200 1.155e-14 ***

2:scale(moon) 0.126002 0.049892 2.5255 0.011553 *

3:scale(moon) 0.468852 0.046884 10.0004 < 2.2e-16 ***

4:scale(moon) 0.446355 0.048040 9.2913 < 2.2e-16 ***

---

Signif. codes: 0 ‘***’ 0.001 ‘**’ 0.01 ‘*’ 0.05 ‘.’ 0.1 ‘ ’ 1

MOST IMPORTANT VARIABLES (absolute value of the t-statistic)

| **Variables Overall**  scale(moon) 1.0413175  scale(nightlength) 0.8132393  scale(Swind) 0.6959832  scale(bathy) 0.4147202 |
| --- |

**Table SI-3.2.a. Adult diurnal behaviour**

Call:

mlogit(formula = lbl ~ 1 | scale(Swind) + scale(bathy) + scale(SLA),

data = ad_day, reflevel = 1, method = "nr", print.level = 0)

Frequencies of alternatives:

1 2 3 4

0.205120 0.098141 0.394697 0.302042

nr method

5 iterations, 0h:0m:0s

g'(-H)^-1g = 1.44E-05

successive function values within tolerance limits

Coefficients :

Estimate Std. Error t-value Pr(>|t|)

2:(intercept) -0.707398 0.070126 -10.0875 < 2.2e-16 ***

3:(intercept) 0.683592 0.049648 13.7688 < 2.2e-16 ***

4:(intercept) 0.419329 0.052039 8.0580 6.661e-16 ***

2:scale(Swind) 0.140840 0.071471 1.9706 0.04877 *

3:scale(Swind) 0.404162 0.050906 7.9393 1.998e-15 ***

4:scale(Swind) 0.375988 0.053184 7.0696 1.554e-12 ***

2:scale(bathy) 0.123259 0.070006 1.7607 0.07829 .

3:scale(bathy) -0.269281 0.050292 -5.3543 8.588e-08 ***

4:scale(bathy) 0.113389 0.052001 2.1805 0.02922 *

2:scale(SLA) -0.120768 0.070572 -1.7113 0.08703 .

3:scale(SLA) -0.245821 0.048651 -5.0527 4.355e-07 ***

4:scale(SLA) -0.232639 0.052538 -4.4280 9.510e-06 ***

---

Signif. codes: 0 ‘***’ 0.001 ‘**’ 0.01 ‘*’ 0.05 ‘.’ 0.1 ‘ ’ 1

MOST IMPORTANT VARIABLES (absolute value of the t-statistic)

**Variables Overall**

scale(Swind) 0.8812630

scale(MSLA) 0.6483631

scale(bathy) 0.5549907

**Table SI-3.2.b. Adult nocturnal behaviour**

Call:

mlogit(formula = lbl ~ 1 | scale(Swind) + scale(bathy) + scale(SLA)

+ scale(moon), data = ad_day, reflevel = 1, method = "nr", print.level = 0)

Frequencies of alternatives:

1 2 3 4

0.28715 0.12384 0.32895 0.26006

nr method

5 iterations, 0h:0m:0s

g'(-H)^-1g = 3.05E-06

successive function values within tolerance limits

Coefficients :

Estimate Std. Error t-value Pr(>|t|)

2:(intercept) -0.8180927 0.0985533 -8.3010 < 2.2e-16 ***

3:(intercept) 0.0487392 0.0779485 0.6253 0.5317910

4:(intercept) -0.1493694 0.0815793 -1.8310 0.0671048 .

2:scale(Swind) 0.1617157 0.1022194 1.5820 0.1136393

3:scale(Swind) 0.3981622 0.0782382 5.0891 3.598e-07 ***

4:scale(Swind) 0.3645740 0.0816516 4.4650 8.007e-06 ***

2:scale(bathy) 0.0133936 0.0974654 0.1374 0.8906996

3:scale(bathy) -0.3564236 0.0758833 -4.6970 2.640e-06 ***

4:scale(bathy) -0.2000303 0.0788071 -2.5382 0.0111416 *

2:scale(SLA) -0.1249952 0.0992831 -1.2590 0.2080383

3:scale(SLA) -0.2649151 0.0753225 -3.5171 0.0004363 ***

4:scale(SLA) -0.2004600 0.0796193 -2.5177 0.0118114 *

2:scale(moon) 0.078232 0.098563 0.7937 0.4273571

3:scale(moon) 0.407227 0.076281 5.3385 9.370e-08 ***

4:scale(moon) 0.376130 0.079888 4.7082 2.499e-06 ***

---

Signif. codes: 0 ‘***’ 0.001 ‘**’ 0.01 ‘*’ 0.05 ‘.’ 0.1 ‘ ’ 1

MOST IMPORTANT VARIABLES (absolute value of the t-statistic)

**Variables Overall**

scale(Swind) 0.9247270

scale(moon) 0.8618476

scale(MSLA) 0.5905468

scale(bathy) 0.5700141
